# The Current Landscape of Adult Neural Stem Cell Research: A Narrative Review

**DOI:** 10.3390/cells15090779

**Published:** 2026-04-25

**Authors:** Jaime Yair Burciaga-Paez, Idalia Garza-Veloz, Margarita L. Martinez-Fierro

**Affiliations:** Molecular Medicine Laboratory, Academic Unit of Human Medicine and Health Sciences, Autonomous University of Zacatecas, Zacatecas 98160, Mexico; burciagapaezjy@gmail.com (J.Y.B.-P.); idaliagv@uaz.edu.mx (I.G.-V.)

**Keywords:** neural stem cells, molecular regulation, neurological diseases, adult neurogenesis, cell culture strategies, stem cell therapy

## Abstract

Adult neural stem cells (NSCs) maintain lifelong neurogenesis, a fundamental process for neuroplasticity, memory and brain homeostasis. Despite decades of research, translating basic NSC biology into effective clinical therapies remains a central challenge. Here we present a narrative review that provides a comprehensive update on the current landscape of adult NSC research, associating molecular mechanisms with the emerging translational technologies. First, we analyze the biological features and neurogenic sequences within canonical niches such as the subventricular lateral zone and the subgranular zone, emphasizing phylogenetic and migratory differences between rodent models and humans. Second, we integrate these mechanisms with the influence of environmental and pathological modulators, describing how aging, metabolic changes, chronic stress and neuroinflammation disrupt NSC quiescence and lineage progression. Finally, we highlight recent technological advances driving the field toward clinical applications. By examining current NSC isolation strategies, induced pluripotent stem cell modeling, direct somatic reprogramming and the use of CRISPR-Cas9-based gene-editing therapies, this review delineates the pathways to overcome existing methodological limitations. Ultimately, we provide an integrated context that connects the modulation of the neurogenic niches with advanced in vitro technologies, offering new perspectives for regenerative medicine and the treatment of neurological disorders.

## 1. Introduction

The acknowledgement that the adult central nervous system (CNS) holds a population of neural stem cells (NSCs) capable of self-renewal and multipotent differentiation has substantially modified traditional views of brain plasticity. Early experimental evidence in rodents demonstrated persistent neurogenesis in specific regions of the adult brain [1], and subsequent studies identified proliferative neural progenitors in the adult human hippocampus [2]. These findings established the presence of specialized neurogenic niches that support ongoing cellular turnover in the mature brain [3,4]. Adult neurogenesis (AN) has been associated with hippocampal-dependent learning and adaptive responses, and alterations in this process have been reported in aging and neurological disorders [5]. Although the extent and functional impact of neurogenesis in humans remain under investigation, accumulating evidence supports its biological relevance under both physiological and pathological conditions [6]. The study of NSCs has therefore become central to understanding cellular plasticity in the adult CNS.

Currently, a significant gap exists between basic NSC biology and the development of effective regenerative therapies for human patients. Despite the presence of resident NSCs, the endogenous repair and regenerative capacity of the adult mammalian brain is limited outside the canonical neurogenic niches [7]. Furthermore, under pathological conditions such as neurodegeneration, severe trauma of the CNS or physiological aging, the microenvironment of the brain becomes hostile [8]. Chronic neuroinflammation, metabolic dysregulation or accumulation of inhibitory extracellular matrix components force NSCs into quiescence and premature exhaustion [9,10].

To harness the regenerative potential of adult NSCs into clinical practice, it is necessary to understand how these cells respond to dynamic modulators so their manipulation becomes effective with the use of modern technologies. Therefore, the primary objective of this study is to address the current translational limitations in the field.

In this narrative review, we establish a comprehensive framework that bridges fundamental biology with emerging therapeutic strategies. First, we delineate the sequence of AN, highlighting crucial phylogenetic differences between rodents and humans. Second, we integrate these baseline mechanisms with the impact of environmental and pathological modulators, such as aging and inflammation. Finally, we explore how recent technological paradigm changes like the refinement of isolation strategies, the application of induced pluripotent stem cell (iPSC) modeling, direct somatic reprogramming and the advent of genome-wide CRISPR-Cas9 for modifying DNA are being deployed to overcome traditional limitations. By articulating these interconnected areas, this review aims to provide a critical, updated perspective on how modulating the adult NSC niche can head for future interventions in neurological diseases and CNS repair.

## 2. Stem Cells in the Adult Central Nervous System

The concept of AN describes the process by which new neural cells, such as neurons, astrocytes or oligodendrocytes, are produced in the CNS during postnatal life [11]. This definition constitutes a shift in a historic paradigm in neuroscience, as the generation of new neurons and cells in the CNS was previously considered not possible beyond embryonic development [12]. There are now known regions of the adult mammalian brain with neurogenic activity, or neurogenic niches, where it is known that AN occurs, providing a microenvironment favorable to the growth and subsequent differentiation of NSCs [13]. The traditional neurogenic niches in adult mammals described are two main brain regions, comprising (1) the subventricular or ependymal zone in the walls of the lateral ventricles (SVZ) [14] and (2) the subgranular zone (SGZ) of the dentate gyrus in the hippocampus (DG) [15]. More recently, the rostral region of the hypothalamus near the third ventricle (HVZ) has also been described as a neurogenic niche [16,17].

**Figure 1:**
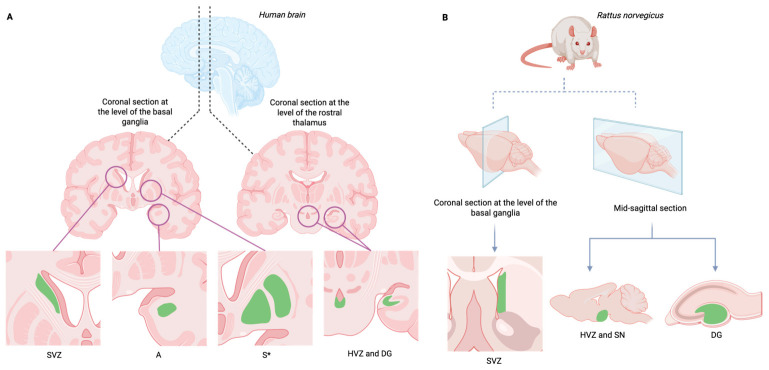
Phylogenetic conservation and spatial divergence of canonical and non-canonical neurogenic niches in the adult mammalian brain. (**A**) In the adult human brain, canonical neurogenesis is major in the subventricular zone (SVZ) in the dentate gyrus (DG) of the hippocampus [2], while the subventricular zone (SVZ) and hypothalamic ventricular zone (HVZ) are down compared to early developmental stages [17,23]. Notably, the striatum and the amygdala serve as confirmed non-canonical neurogenic niches in humans [19,20]. (**B**) In contrast, the adult rodent brain exhibits a more robust and widespread neurogenic capacity. Alongside highly active canonical niches such as SVZ, SGZ and HVZ, rodents maintain other non-canonical neurogenic regions, including the substantia nigra. Abbreviations: SVZ, subventricular zone of the lateral ventricles; A, amygdala; S, striatum (* globus pallidus on the left, putamen on the right); HVZ, hypothalamic ventricular zone; DG, dentate gyrus of the hippocampus; SN, substantia nigra.

Although the first descriptions of cell migration and proliferation were made in the hippocampus [1], where a higher volume of results suggestive of AN was reported, in recent years new neurogenic niches have been described as non-canonical niches [18]. As illustrated in Figure 1, while the localization of canonical neurogenic niches such as the SVZ and the SGZ is conserved across mammalian species, phylogenetic differences exist regarding non-canonical niches. These niches involve brain locations in humans such as the striatum [19] and the amygdala [20] (Figure 1A) and the substantia nigra pars compacta in rodents [21,22] (Figure 1B).

The biological differences in the mechanisms and dynamics of AN between mammals are significant, requiring caution when translating findings from animal models to humans. While rodent models have provided foundational knowledge regarding the multipotent nature of NSCs, significant phylogenetic differences exist. For instance, neurogenesis in the SVZ is considerably reduced after infancy in adult humans, compared to sustained neurogenesis in this niche in rodents throughout life [23]. Therefore, throughout this review, findings derived from specific animal models are explicitly indicated to provide accurate scientific context.

### 2.1. Characteristics of Neural Stem Cells and the Sequence of Adult Neurogenesis: Proliferation, Differentiation, Migration and Maturation

The term NSCs refers to the populations of stem cells that reside within specialized neurogenic niches in the adult. These cells are defined by two main characteristics: their potential for multipotent differentiation and their capacity for long-term self-renewal [13]. Rather than showing pluripotency, adult NSCs maintain a lineage-restricted multipotent state that is regulated by the local microenvironment, giving rise to three main lineages of the CNS: neurons, astrocytes and oligodendrocytes.

During the process of AN, these NSCs, which are usually dormant, become active and undergo asymmetric division, generating highly proliferative neural progenitor cells (NPCs) that subsequently progress through targeted differentiation, migration and final functional maturation [24]. Consequently, AN is regulated by specific processes and presents with distinct cellular and molecular characteristics depending on the region in which it occurs [25]. Therefore, the neurogenesis process must be delimited for SVZ and SGZ separately.

#### 2.1.1. Proliferation, Cell Fate Determination and Differentiation

**Figure 2:**
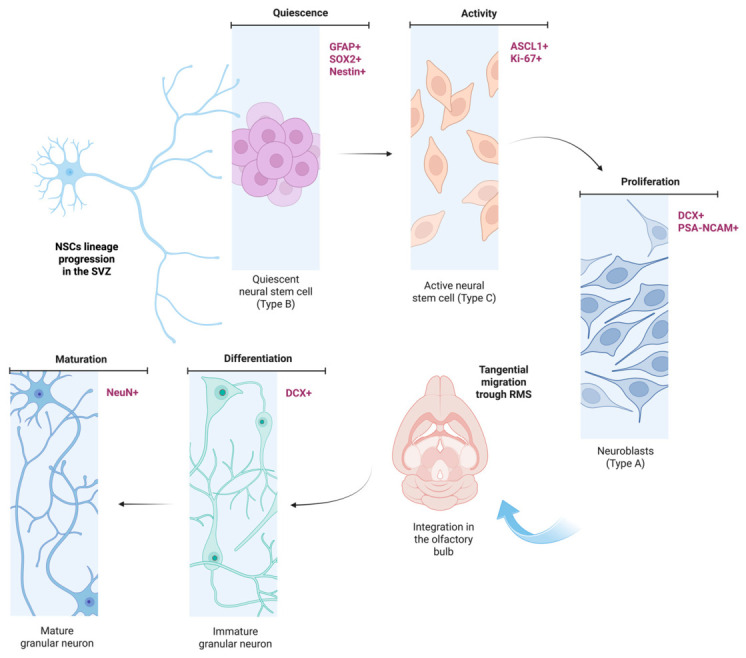
Lineage progression and sequential molecular restriction during adult SVZ neurogenesis in rodents. The neurogenic cascade in the subventricular zone (SVZ) involves a coordinated transition across distinct cellular stages: quiescence, activation, proliferation, migration, differentiation and maturation. Quiescent NSCs (Type B cells, expressing GFAP+/SOX2+/Nestin+) [31,32] become activated and divide asymmetrically to generate transit-amplifying cells (Type C cells, expressing ASCL1+/Ki-67+) [27]. These progenitors commit to a neuronal fate, giving rise to immature neuroblasts (Type A cells, expressing DCX+/PSA-NCAM+) [28]. Neuroblasts then assemble into chains and migrate tangentially through the rostral migratory stream (RMS) toward the olfactory bulb (OB). Upon arrival, they detach from the migratory chain, mature and functionally integrate as local interneurons, defined by the expression of mature markers (NeuN+) [33]. Abbreviations: NSCs, Neural Stem Cells; SVZ, Subventricular Zone; ASCL1, Achaete-scute homolog 1; DCX, Doublecortin; GFAP, Glial Fibrillary Acidic Protein; SOX2, SRY-box 2; NeuN, Neuronal nuclear antigen; PSA-NCAM, Polysialylated-Neural Cell Adhesion Molecule.

In the SVZ in rodents, quiescent NSCs known as Type B cells become activated by microenvironment cues and undergo asymmetric division to give rise to transit-amplification cells, or Type C cells [26]. These Type C cells are highly proliferative and are distinctively characterized by the expression of the proneural Achaete-Scute transcription factor of the BHLH family 1 (ASCL1, or Mash1) [27]. The expression of this factor is fundamental in the SVZ, since these progenitors are predominantly set to generate inhibitory neuronal lineages. Subsequently, Type C cells differentiate into neuroblasts (Type A cells), which express Doublecortin (DCX) [28]. Unlike their hippocampal equals, SVZ neuroblasts undergo extensive tangential migration through the rostral migratory stream (RMS) to reach the olfactory bulb (OB), where they finally mature into inhibitory interneurons and integrate into local olfactory circuits [29,30]. As presented in Figure 2, Type B cells are characterized by the expression of classical glial and stemness markers, including glial fibrillary acidic protein (GFAP), SRY-box 2 (Sox2) and Nestin [31,32], while Type C and Type A cells express markers associated with their function, such as cell cycle markers in the case of Type C cells and cytoskeletal remodeling and migration markers in the case of Type A cells. Upon integration into preexisting circuitry, neuroblasts undergo terminal differentiation, expressing mature neuronal markers such as neuronal nuclear antigen (NeuN) [33].

Conversely, neurogenesis in the SGZ in rodents follows a local, functionally distinct trajectory aimed at producing excitatory neurons [34]. In this niche, radial glia-like NSCs (Type 1 cells) divide to generate intermediate progenitor cells (Type 2 cells) [35]. In contrast to SVZ Type C cells, Type 2 cells are characterized by the expression of the T-box brain protein 2 (TBR2) [17]. The expression of TBR2 is a specific molecular hallmark of progenitors committed to an excitatory glutamatergic lineage [36,37]. Then, TBR2-positive Type 2 cells differentiate into DCX-positive neuroblasts (Type 3 cells) [11]. Migration in the SGZ is strictly radial and covers a shorter distance than its counterparts in the SVZ. Neuroblasts migrate into the adjacent granule cell layer of the DG, where they mature into excitatory glutamatergic granule neurons, essential for hippocampal-dependent memory and cognition [38].

In the adult rodent hypothalamus, tanycytes and ependymal cells are identified by the expression of genes encoding Sox2, Nestin, glutamate-aspartate transporter (GLAST) and GFAP [16,17]. Pioneering lineage-tracing studies have demonstrated that distinct subpopulations of these tanycytes showed a particular ability to participate in local networks of hypothalamic neurons and in the roles of energy balance and regulation [39]. This demonstrated that the HVZ is sensitive to systemic metabolic cues, unlike SVZ and SGZ niches.

#### 2.1.2. Direct Migration and Navigation

At the end of the 20th century, the description of the migration of new neural cells to the OB, the corpus callosum, the neocortex and the striatum in adult rodents was consolidated [40,41]. The RMS is the predominant migratory route and represents a pathway through which derived neuroblasts from the SVZ migrate tangentially to reach the OB, where they integrate as local interneurons [30]. This migration is organized into a chain formation, where cells move collectively without the need for radial glial guides [42]. The processes of the SVZ astrocytes act as glial tubes that restrict and modulate the migratory path of cells [43]. Furthermore, migrating neuroblasts use local blood vessels running parallel to the RMS as a physical scaffold. At the molecular level, this chain migration is maintained by homotypic cell–cell interactions mediated by the polysialylated neural cell adhesion molecule (PSA-NCAM) [44]. Chemoattractants such as blood-vessel-derived brain-derived neurotrophic factor (BDNF) signal directionality and pace of migration for these cells, and repulsive cues such as the secretion of Slit1 by the Slit-Robo pathway from neuroblasts repel astrocytic processes, clearing their migratory path [45].

In contrast to rodents, the migratory pathways in humans exhibit profound differences in both destination and lifespan. During postnatal development, human neuroblasts migrate through a unique medial migratory stream and route young neurons into the adjacent subcortical white matter and the ventromedial prefrontal cortex by a distinct “Arc” structure [46]. Histological characterization of the adult human SVZ reveals an astrocyte ribbon that lacks the classic chain migration architecture [47]. Consequently, while the fundamental molecular machinery of motility might be conserved, the structural pathways and the neurogenic output scaling differ. While continuous chain migration directed to the OB is a hallmark of the adult rodent brain, human migration is massive but temporally restricted to early infancy and spatially redirected towards cortical regions of higher order [23].

#### 2.1.3. Functional Maturation and Synaptic Integration

Specific transplantation of NSCs to different sites in the CNS has been found to lead to different cell functional maturation, perhaps influenced by the microenvironment, as it has been shown that cells implanted in the spinal cord of rats end up becoming cells that are different from those introduced into the granular layers and can differentiate into specific cell lines that meet the characteristics of the region, such as astroglia and oligodendroglia cells [48]. However, it has been observed that this delineation is not merely a result of the environment in which the cell populations are found but also because of the cell line, raising the possibility of a difference between the various types of stem and progenitor cells in different sites of the adult mammalian brain, as well as in the developing brain during the embryonic stage [49]. Thus, it has been proposed that the stemness state should be perceived as a broad and flexible phenotype rather than a cell type. This perspective better explains the complex regulatory mechanisms of neurogenesis, given that cells within the CNS proliferative niches exhibit significant transcriptional heterogeneity [50]. Current studies based on this premise examine the heterogeneity of cells. For example, Cebrian-Silla et al. demonstrated the existence of two distinct populations of neural stem and progenitor cells in the dorsal and ventral domains of the SVZ in murine models, expressing Crym and Urah/Dio2, respectively, for these regions [51]. This line of research has demonstrated that heterogeneity in NSC is related to their reproductive capacity, as populations of NSCs marked by Gli1 possess long-term self-renewal capacity exceeding 100 days, while cells marked by ASCL1 are reduced after activation [52]. These findings demonstrate that Gli1-positive cells show abundance in symmetrical duplicative divisions and can return to a state of quiescence after producing progeny compared to other NSCs [52].

The multipotency of adult NSCs and their sequential restriction toward neuronal or glial fates are encoded at the transcriptional level, as summarized in Table 1. Rather than a fortuity process, lineage commitment requires a highly coordinated shift in molecular profiles. The transition from multipotent NSCs to mature neurons involves the sequential downregulation of stemness factors (SOX2 and Nestin) and the upregulation of proneural and migratory markers like ASCL1 and DCX [53,54]. At the same time, the gliogenic potential of NSCs is evidenced by divergent transcriptional trajectories. The oligodendrogenic cascade is regulated by the persistent expression of Olig2 in highly proliferative oligodendrocyte precursor cells (OPCs), which subsequently express myelin-associated proteins like MOG upon terminal maturation [55]. Similarly, the astroglial lineage is defined by the acquisition of mature metabolic and structural markers, such as S100β, establishing a clear molecular boundary from the progenitor state [56].

#### 2.1.4. Astroglial and Oligodendroglial Lineages

Although NSCs are recognized for their neurogenic capacity, they also continuously generate astrocytes and oligodendrocytes to maintain CNS homeostasis and facilitate repair. The transition toward an astroglial fate seems driven by specific microenvironmental signals, such as bone morphogenetic proteins (BMPs), which actively promote astroglial lineage commitment by progenitors from the SVZ [58]. During astrogliogenesis, progenitors upregulate mature glial markers, including GFAP and S100β [60]. In the adult rodent hippocampus, this astrogliogenic process is inherently coupled to NSC division, eventually leading to terminal astrocytic lineage and the age-related depletion of active NSCs [56].

Equally, the oligodendrogenic pathway involves the generation of OPCs [59]. The acquisition of this fate is directed by specific transcription factors such as Olig2 [55]. These adult-born OPCs express NG2 and PDGFRα surface markers and migrate from the SVZ into adjacent parenchymal regions and white matter tracts, such as the corpus callosum [59]. Upon reaching their destination, they terminally mature as oligodendrocytes to form new myelin sheaths. Importantly, under pathological conditions such as demyelinating lesions (like multiple sclerosis), this endogenous gliogenic program is dramatically amplified, mobilizing resident neural progenitors to undergo massive oligodendrogenesis to repair damaged tissue [61].

Beyond the CNS, the gliogenic plasticity of adult NSCs is currently being harnessed for peripheral nervous system repair. When implanted into bioengineered nerve conduits, NSCs exhibit the capacity to differentiate and mature into myelinating Schwann cells, a process that provides a growth-factor-enriched microenvironment critical for restoring damaged peripheral nerves [62]. This specific glial commitment is modulated by immune-derived cytokines. Specifically, the cytokine IL12p80 serves as a potent extrinsic inducer that triggers Schwann cell differentiation from NSCs via the phosphorylation of the Signal Transducer and Activator of Transcription 3 (Stat3) pathway. In vivo models have demonstrated that the synergistic implantation of NSCs and IL12p80 into sciatic nerve lesions significantly enhances the generation of mature Schwann cells and increases the diameter of regenerated axons, accelerating functional motor recovery [62]. As highlighted by Lee et al., understanding this gliogenic potential is critical, as restoring the proper glial microenvironment is as essential as neuronal replacement in treating severe neurodegenerative and demyelinating diseases [62].

### 2.2. Modulatory Factors of Neural Stem Cells

**Figure 3:**
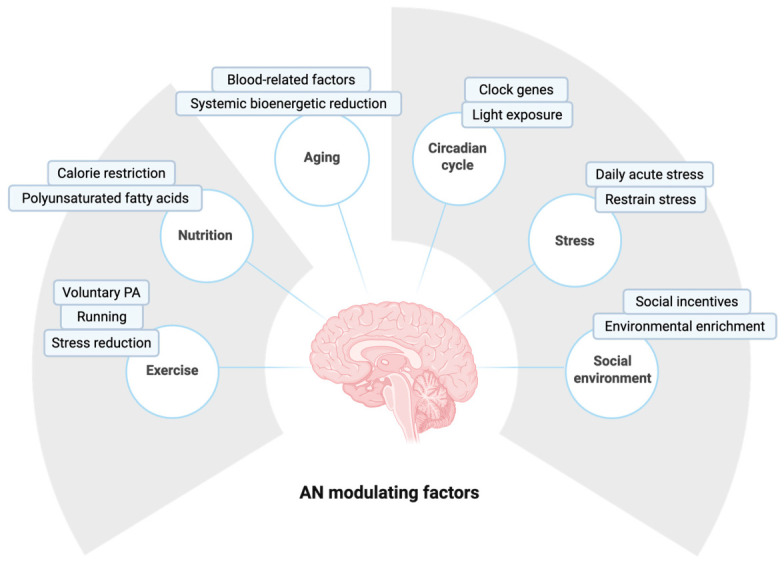
The dynamic modulation of AN by systemic, environmental and behavioral factors. The adult neurogenic niche is highly responsive to both extrinsic and intrinsic cues. Positive lifestyle interventions, including voluntary physical exercise, social enrichment and balanced nutrition, actively upregulate NSC proliferation, differentiation, and newborn neuron survival [63,68]. In contrast, adverse physiological and environmental conditions, such as chronic stress, disruptions in the circadian cycle and biological aging, establish an inhibitory microenvironment [65,66]. These negative modulators induce stem cell quiescence, promote neuroinflammation and accelerate the age-related depletion of the neurogenic pool. The homeostatic balance between these opposing forces determines the lifelong trajectory of hippocampal plasticity and cognitive health. Abbreviations: AN, Adult Neurogenesis; PA, Physical Activity.

AN is sensitive to a wide range of systemic, environmental, and behavioral cues in the adult human brain and in other species. As summarized in Figure 3 and described throughout this subsection, neurogenic niches continuously integrate internal and external stimuli to adapt structural brain plasticity to physiological demands. Positive modulators, such as voluntary physical exercise [63], social and environmental enrichment [41] and specific nutritional interventions [64], promote NSC activation, enhance progenitor proliferation and secure the survival of newborn neurons. Conversely, intrinsic age-related metabolic decline [65], circadian rhythm disruptions [66] and chronic stress [67] create a hostile systemic environment. These negative modulators drive NSCs into deep quiescence, promote neuroinflammation or lead to premature exhaustion. Understanding the interplay of these modulatory factors provides a foundation for developing non-pharmacological and targeted therapeutic interventions aimed at preserving cognitive resilience and combating neurodegenerative diseases.

#### 2.2.1. Aging

Reductions in the number of NSCs have been observed in older animal models compared to younger models, indicating that age plays an important role in the proliferation rate and number of cells involved in AN [69]. In a study conducted by Puvogel et al. [70], 11 cell clusters related to NSCs, including neuroblasts and oligodendrocyte progenitors, were compared in post-mortem tissue samples from the sub-ependymal region of young people aged 16 to 22 years against those of adults aged 44 to 53 years. They did describe a reduction in the quantity of oligodendrocyte progenitors and microglia in the adult group, as well as a decrease in the expression of genes related to NCS development, but the neuroblast clusters remained expressed, suggesting a continuation of the proliferation of these cells despite age in humans [70]. In a similar way, Boldrini et al. [71] studied the postmortem brains of healthy humans aged between 14 and 79, observing that neurogenesis persists in old age, maintaining stable numbers of progenitors and immature neurons, although with reduced niche plasticity [71].

Heterochronic parabiosis has been a key experimental technique for studying the influence of the systemic environment on brain aging and neurogenesis, which consists of surgically connecting the circulatory systems of young and aged animal models [72]. Thus, it has been observed that exposure to aged blood inhibits progenitor cell proliferation in the DG and SVZ of young mice, while exposure to young blood rejuvenates proliferation and neuronal differentiation in older animals. The identification of pro-aging factors in blood such as beta-2-microglobulin (B2M) and eotaxin-1 (CCL11) has been described by studying different analytes in blood plasma, establishing an inhibitory effect on AN by systemic or local administration [73]. Similarly, Buckley et al. [74] demonstrated that heterochronic parabiosis and exercise reverse transcriptomic aging in NSCs in mice, as parabiosis reverses interferon response genes, while exercise reduces the expression of Dbx2, related to the decline of neurogenesis in the SVZ [74].

The decline in neurogenesis with age could also be due to mitochondrial protein folding stress mediated by sirtuin 7 (SIRT7), as described in a study published by Wang et al. [75]. In their study, the use of single-cell RNA sequencing (scRNA-seq) in the DG showed that such stress increases in NSCs during aging, compromising the maintenance of these cells and leading to cell cycle dysregulation [1].

It is known that age in relation to AN also appears to be modulated by microglia. This finding comes from a study conducted in 2012 using transgenic mice deficient in a gene encoding chemokine receptor in microglia cells, the CX3CR1, compared to wild-type mouse models [76]. It was demonstrated by fluorescence-activated cell sorting or flow cytometry (FACS) that microglia mediate the effects of exercise on NSC activity, and, in aged mouse models, these cells contribute to the suppression of NSCs [76].

Recent studies have also highlighted the crucial role of metabolism in NSC aging. Ruetz et al. demonstrated, using CRISPR-Cas9 screening, that the knockout of Slc2a4 significantly improves the function of aged NSCs [77]. Rather than experimental overexpression, their work revealed that old NSCs naturally exhibit increased glucose uptake with age, and that the targeted knockout of Slc2a4 effectively restores the capacity of aged NSCs to activate and generate new neurons in vivo and in vitro [77]. According to Silva-Vargas et al. [78], the lateral ventricle choroid plexus (LVCP) in the SVZ acts as an underrated region in neurogenesis due to the secretion of modulatory factors of NSCs that also vary with age, negatively modifying the molecular environment [78]. Thus, factors such as BMP5 and insulin-like growth factor type 1 (IGF1) appear to enhance cellular activity, positioning the LVCP as a key metabolic and sensory axis for brain health and repair [78].

Emerging evidence indicates that age-related decline in AN is sexually dimorphic, since advanced 3D imaging of the entire SVZ niche has revealed that male and female mammalian brains exhibit different trajectories during physiological aging [79]. Specifically, aging males experience deterioration of the vascular niche accompanied by a depletion of quiescent NSCs and disorganized neuroblast migration, while in females, the population of apical NSCs and overall progenitor proliferation remains stable [79].

#### 2.2.2. Physical Activity and Exercise

Physical activity, especially voluntary exercise, has been shown to be a powerful modulator of A. The relationship between AN and exercise may be present due to a general balance coordinated via cells such as astrocytes [80]. Exercise appears to significantly increase the proliferation of progenitor cells in the SGZ, as well as their survival and maturation rates [81]. This effect is consistently observed in rodents housed with running wheels and in physically enriched environments. Physical activity positively correlates with enhanced progenitor cell proliferation, survival and maturation. These changes translate into improved cognitive performance, including better spatial memory and pattern discrimination, as well as reduced anxiety and depression [80]. Studies have also shown that in mouse models voluntary exercise mitigates behavioral alterations caused by chronic restraint stress. At the cellular level, exercise restores the activity of serotonin 5-HT1A receptors in hippocampal granule cells, which are typically diminished by stress. Additionally, it promotes indirect modulation through 5-HT3 receptors, restoring GABAergic inhibition and enhancing stress resilience [82]. Exercise also promotes dendritic growth, spine formation, and neuronal integration in newborn cells [80]. Other studies reveal that voluntary running not only increases the survival of neurons created in youth but also modifies the synaptic connectivity network between them in the hippocampus and cerebral cortex. Using rabies viral tracing, Vivar et al. [83] demonstrated in mice that long-term exercise affects neurons that are originally born during youth [83]. They found that exercise preserves and remodels the connectivity of postnatally created neurons. Specifically, they found that running prevented the loss of innervation from the perirhinal cortex and increased afferents from the caudomedial entorhinal cortex, the subiculum, and local inhibitory interneurons, improving connectivity for memory function in middle age [83]. One mechanism that could influence this phenomenon is seen through the VEGFR2/Flk-1 signaling pathway, since blocking of this signaling also inhibits the neurogenic benefits of exercise [84]. Furthermore, voluntary exercise appears to be able to counteract the natural decline in NCS activity that occurs with aging by also slowing cognitive decline. Even in older rodents, physical activation has been found to improve learning and neogenesis in the CNS [76]. Buckley et al. [74] found that using scRNA-seq and aging clocks, exercise has a specific rejuvenating effect on the SVZ, as it reversed the biological age of oligodendrocytes and NSCs. However, this effect was less pronounced in the SVZ compared to the heterochronic parabiosis described above [74].

Adaptive plasticity in the adult CNS relies on different mechanisms, ranging from structural glial responses following injury to dynamic neuronal synchronization during learning. On one hand, following a peripheral lesion such as unilateral vestibular neurectomy, an acute proliferation of microglia is activated within the deafferented vestibular nuclei. This intense localized glial plasticity is proposed to act as an intrinsic, adaptive biological mechanism that facilitates functional vestibular compensation [85]. On the other hand, functional plasticity at the circuit level is equally essential for cognitive processing. In the murine DG, mature granule cells generate synchronized population activity during periods of behavioral immobility. Driven by cortex inputs, these activity torrents summarize the neural patterns evoked during previous spatial exploration. Interestingly, the specific inhibition of this synchronized DG activity during rest prevents the consolidation of precise spatial memories [86].

#### 2.2.3. Nutrition

Diet and nutritional factors can influence the production of new neural cells in adulthood, as research has revealed that both the type and timing of food intake can modulate neurogenesis with significant implications for brain health and mental function. The nutritional factor associated with AN stems from evidence that polyunsaturated fatty acids and other compounds present in food improve this process in mice [87]. These compounds can enhance AN by modifying the cell membrane and improving the binding of serotonin to its brain receptors or by regulating neurotrophin levels such as BDNF. At the same time, negative effects have been described for diets high in fat and refined sugars, known as high-calorie diets (HCDs), which impair AN least in the hippocampus [68]. This is because HCDs have been associated with reduced cell proliferation, decreased neuroblasts and mature neurons, and memory dysfunction in response to neuroinflammation [88]. Leiter et al. [89] revealed in a study with aged mice that systemic administration of selenium reversed the decline in neural precursor proliferation by mimicking the effects of exercise, improving neurogenesis. The process reduces cellular oxidative stress (OS) and promotes neuron proliferation, leading to improved cognitive function and spatial memory [89].

Calorie restriction and intermittent fasting have been shown to promote AN, as clinical trials and observational studies have evaluated the impact of certain diets that restrict eating times on health markers, risk factors for age-related diseases, and cognitive function [87]. Furthermore, this restriction seems to increase the number of neuroblasts, the survival of NSCs, and their differentiation into mature neurons [88]. At the same time, acute fasting decreases the proliferation of these cells, while refeeding increases it. This control is mediated by neuronal innervations that originate in the hypothalamus and reach the SVZ niche [90].

Lipid metabolism is closely associated with neurogenesis, as it has been found that the proliferation of adult NSCs depends on de novo lipogenesis mediated by the enzyme fatty acid synthase (FASN). Its elimination stops the production of new brain cells, while the expression of the Spot14 protein keeps them quiescent [91]. These findings correlate with the fact that the R1812W mutation in FASN causes a toxic accumulation of lipids in NSCs, inducing endoplasmic reticulum stress and cognitive defects in mouse models [92].

Kandel et al. [93] explored oleic acid, a monounsaturated fatty acid, which appears to act as an endogenous ligand for the TLX receptor (NR2E1). Oleic acid binds to TLX, converting it from a transcriptional repressor to an activator of cell cycle and neurogenesis genes [93]. The authors observed that after injecting exogenous oleic acid into the DG of mice of different ages, proliferation of NSCs and the generation of new neurons increased, reversing deficits associated with aging [93]. Furthermore, Ramosaj et al. [94] demonstrated that NSCs accumulate fat storage organelles called lipid droplets and that a greater number of them improves cell proliferation capacity in adult mice. Artificial loading of lipids with oleic acid prior to cell differentiation improved neuron generation, while blocking the degradation of these droplets reduced their proliferation, suggesting that NSCs need access to these energy reserves to divide efficiently. Manipulating the accumulation or breakdown of these lipids can directly alter neurogenesis, suggesting once again that fat metabolism is essential for maintaining the reserve of neurons and protecting stem cells from oxidative damage [94].

In a longitudinal study in humans combined with in vitro assays using participants’ serum, low levels of prealbumin were associated with increased apoptosis. Elevated levels of β-cryptoxanthin and low-density cholesterol were associated with improved integrity and maintenance of SOX2-labeled progenitor cells [95]. Furthermore, treatment with phosphatidylcholine, a major structural phospholipid component of neuronal membranes, was shown to promote neuronal differentiation of NSCs even under conditions of inflammatory stress in mice. Phosphatidylcholine improved the number of neurons and their functionality [96].

#### 2.2.4. Stress and Social Environment

Stress appears to play an important role in neurogenesis, as both long-term stressful environmental conditions and acute and chronic stress cause a decrease in neural cell proliferation [97]. Tests performed on rodents using mazes like Morris Water or Elevated Plus Maze have been fundamental to describe changes in neurogenesis associated with stress. Significant growth in the population of progenitor cells in the DG of the hippocampus was observed in adult rodents exposed to a stimulus-enriched environment, compared to groups of rodents deprived of stimuli [41]. The participation of individuals in a stimulus-rich environment is also associated with a decrease in anxiety-related behaviors while stimulating emotional reactions that positively influence hippocampal neurogenesis and stress reduction [2]. The vascular endothelial growth factor (VEGF) is reduced in socially isolated mice and with induced depression, consequently presenting a decrease in AN [67]. Also, unpredictable chronic moderate stress is associated with increased microglia and astrocyte activation in the ventral and dorsal regions of the DG. In this study, young adult mice were exposed for 15 weeks to a protocol of isolation and chronic unpredictable moderate stress. Subsequently, the behavior of the models was evaluated, as well as the postmortem biochemical and histological parameters of their brain tissues. The results showed dystrophic morphology in the ventral DG of the hippocampus, among other findings related to depression. Cell proliferation and differentiation in the DG of the hippocampus were also found to be reduced, although no alterations in its structural volume were found [67].

Studies have shown through RNA interference (RNAi) of neuropeptide Y1 receptor (NPY1R) the disarticulation of heteroreceptors such as GALR2 and TrkB in rats, but not modified neurogenesis or depressive behaviors shown in short-term models. This suggests that there are strong compensatory mechanisms against acute disruptions in resilience signaling [98].

Interactions in different mammalian species during adulthood in relation to their social environment, such as reproduction, social status, social integration and parenthood, seem to have a direct relationship with the ability to generate new neural cells. At the same time, isolation and social defeat have a negative effect on NSC proliferation [99]. Interestingly, chemogenetic inhibition of new neurons increases susceptibility to social defeat stress, while increasing neurogenesis in the ventral area promotes resilience [100]. During pregnancy, a reduction in antepartum cortical volume has been established in women, while brain tissue appears to recover at different rates after birth, depending on the region and type of delivery experienced [101]. These findings are related to a longitudinal study of primiparous and nulliparous women where it was found that greater perceived stress during the transition from pregnancy to postpartum is associated with symptoms of depression and lower maternal bonding. These psychological stressors negatively influenced brain plasticity and the behavioral adaptation necessary for parenting [101]. On the other hand, the presence of interneurons related to pregnancy has also been demonstrated with the activation of cell markers that are usually quiescent and, at different stages of this period, are activated [102].

## 3. Conserved Molecular Mechanisms in Embryonic and Adult Neural Stem Cells

While the neurogenic niches of the adult mammalian brain differ significantly from the developing embryonic environment, NSCs share a common cell lineage with embryonic radial glial cells (RGCs). Recent single-cell RNA sequencing studies have revealed that the transition of adult NSCs from a quiescent state to an active state involves the awakening of developmental programs that originally operate in embryonic RGCs [103]. Consequently, classic morphogens and signaling pathways responsible for embryonic patterning and neurogenesis such as Notch, Wnt, Sonic Hedgehog (SHH), and BMPs are critically conserved in the adult CNS [58]. However, in the adult neurogenic niches, these developmental pathways are uniquely adapted to regulate the balance between stem cell quiescence, proliferation and differentiation.

The factors involved in the regulation of AN and the molecules that modulate it have been the focus of study in recent years, revealing important evidence about their close relationship with the body’s physiological mechanisms and, in turn, with important pathological processes. Diverse neurotrophic growth factors, including BDNF, VEGF and fibroblast growth factor 2 (FGF2), activate downstream intracellular cascades that dictate the delicate balance between deep quiescence, active proliferation and survival [104]. Table 2 provides an integrated overview of these critical molecular factors, highlighting their specific functional roles across the neurogenic cascade.

As discussed before, AN is rhythmic and closely related to the circadian cycle and the factors that regulate it [115]. An example of this rhythm is highlighted by the absence or dysfunction of clock genes, for example, by deprivation of the central clock gene of the brain and muscle aryl hydrocarbon receptor translocator (Bmal1) [115]. This gene is related to a reduction in NSCs in the DG and an increase in the activation of mechanisms related to OS and senescence. The absence of Bmal1 itself causes the activation of Sirtuin 1 (SIRT1), which ultimately reduces AN and at the same time promotes the senescence process, as well as having effects on the migration of NSCs [115]. In turn, cell loss in hippocampal structures does not cause alterations in their volume, reflecting that there are only changes in cellularity.

The clock mechanism has also been suggested by the circadian regulator 1 (PER1) gene and its diurnal expression in the DG [116]. This rhythmicity in AN has also been evidenced by its dependence on light, in relation to the times of day when cells proliferate and enter a cycle of mitosis. These findings were made in rodents in the DG of the hippocampus, while studies on the SVZ showed greater proliferation during the day, demonstrating that neurogenesis is not only related to temporality but is also dependent on the brain region [117].

One of the first molecules found to be related to NSC proliferation is FGF2. In a study published in 1987 [118], 13-day-old rat embryos were placed in a culture rich in this factor for different periods of time. The cultures managed to survive for five days. In this type of study, autoradiography and the marking of cells incorporating tritiated thymidine were also used, demonstrating that FGF2 was a mitogenic factor in CNS neural precursor cells in rats in vitro [118]. Other growth factors such as epidermal growth factor (EGF) and transforming growth factor alpha (TGF-α) are associated with the differentiation process of neural cells in the postnatal development of the retina in mammals [119]. Differentiation has been observed in neuroepithelial cells with modification of their phenotype to glial cells and neurons specific to the retina, suggesting these factors as strong mitogens in this process [119]. The discovery of brain EGF present in astrocytes and neurons was significant for its subsequent association with the development of these and other mature cells. EGF has also been linked to neuron-glia communication [120]. EGF is also related to the survival capacity of the proliferation process in CNS neuron cultures in newborn rodents, which shed light on the function of this factor as an aid in neurite elongation and maintenance [121]. Other genes that have been associated with AN are BDNF and neurotrophin-3 (NT-3), as well as nerve growth factor (NGF) in cell proliferation using the bromodeoxyuridine (BrdU) method [81,122]. NGF was found to promote the survival and growth of neurites in neurons of the central and peripheral nervous systems in vertebrates. In addition, Nestin expression is used as a marker for cells with proliferative and precursor activity [122]. Research into the role of the protein homologous to phosphatase and tensin (PTEN) in the long-term maintenance of RGCs has shown its positive influence on their functions, including the maintenance of the quiescent state, self-renewal, and their differentiation into astrocytes [11].

It was proposed that IGF1 could be involved in regulating the migration process of NSCs in relation to hormonal control [123]. Microglia secrete factors such as tumor necrosis factor-alpha (TNF-α), interleukin-1 (IL-1) and IGF1 that promote the differentiation of NSC into dopaminergic neurons and regulate the survival of progenitor cells [124]. Factors such as SOX9 and NFIA are important for the initiation of gliogenesis and the repression of neurogenesis in later stages [125]. Thus, EBF1 and ISL1 drive differentiation toward striatal neurons, while other programs direct differentiation toward the OB [126]. Signal trafficking via extracellular vesicles (EVs) participates in brain structure organization, migration and differentiation [127]. Domingo-Muelas et al. [128] demonstrated in adult mice that the MEX3A protein controls a signature of transcripts associated with quiescence, acting as a translational remodeler [128]. The study revealed a dose-dependent dual function: low levels of the protein allow NSC to return to a resting state to maintain long-term cell reserve, while its elevation is necessary to repress this genetic program and allow neuroblasts to differentiate [128]. Marques-Torrejón et al. [129] identified the transmembrane protein LRIG1 as a regulator of NSC quiescence using CRISPR-Cas9 screening, as it inhibits epidermal growth factor receptor (EGFR) signaling, preventing disorganized division. They also suggest that a primed state induced by the combination of BMP and FGF signals would allow cells to be ready to reactivate quickly [129]. The activation of this state has been associated with the ability to detect peripheral inflammatory signals, such as those caused by a distant injury, facilitating a faster regenerative response. These findings are demonstrated using single-cell transcriptomics and mouse models, together with the identification of TNF-α as a key molecular regulator with a dual function: while the TNFR2 receptor promotes the activation and self-renewal of NSCs, the TNFR1 receptor favors the maintenance of deep latency [109].

Recently, Ciceri et al. [130] discovered that certain chromatin factors function as an epigenetic barrier that keeps maturation programs in a latent state. They demonstrate that transient inhibition of EZH2 or DOT1L in progenitor cells allows the resulting neurons to rapidly achieve mature functional and transcriptomic properties [130]. Certain transcription factors regulate cell differentiation, such as OLIG2, which suppresses other destinies and promotes the development of motor neurons by inducing Neurogenin 2 (NGN2) [131]. NGN2 negatively regulates the paired box 6 (Pax6) gene and ASCL1 to enable cortical neuronal differentiation [132].

Thyroid hormones appear to play a role in balancing progenitor proliferation and differentiation into neural lineages in the adult SVZ. These hormones are in turn regulated by transporters such as MCT8 and OATP1C1 [133]. Other factors related to the AN process in mammals are microRNAs (miRs), neurotransmitters, and neurotrophins, as the expression of the latter by macrophages in the brain microglia is related to the proliferation of this same tissue [134]. The release of neurotransmitters by neuroblasts, such as gamma-aminobutyric acid (GABA), reflects the mediation of neurotransmitters in AN, as does the release of glutamate by astrocytes. This is intrinsically related to the multifactorial nature that favors neurogenesis and the various mechanisms by which it could be altered [135]. Other factors such as methyl-CpG and MBD1 are described as regulators of proliferation and differentiation through the control of specific miRs, such as miR-184 [136].

## 4. Current Isolation Strategies and Cell Culture of Adult Neural Stem Cells

In addition to providing structural organization, the cells that compose the adult brain actively interact to regulate homeostasis, coordinate signaling and mediate responses to injury or disease. Consequently, the functional and organizational complexity of the CNS is difficult to replicate, and this challenge becomes even greater under pathological conditions [137]. To accurately investigate the fundamental properties of adult NSCs, like their self-renewal capacity, multipotency and response to modulatory factors, it is essential to extract them from the complex niche in which they reside and propagate them in highly controlled in vitro systems [138]. Therefore, developing robust isolation strategies and optimized cell culture protocols has become invaluable in modern neurobiology, providing the necessary platforms to scale up NSC populations for both basic research and potential regenerative therapies.

### 4.1. Strategies for Obtaining NSCs in Humans

The main strategies for obtaining human NSCs can be currently classified into three fundamental methodological groups: (1) direct isolation from primary fetal or adult CNS tissues, including the extraction from classic neurogenic niches, the spinal cord or the olfactory mucosa; (2) directed differentiation from pluripotent stem cells, such as embryonic stem cells (ESCs) or iPSCs; and (3) direct lineage reprogramming or transdifferentiation from somatic cells.

**Figure 4:**
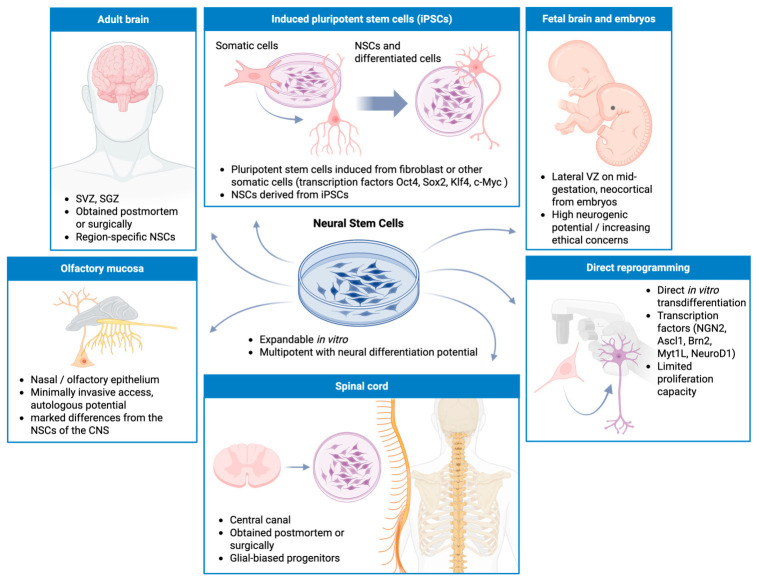
Schematic overview of the principal biological sources and experimental strategies used to obtain neural stem cells (NSCs). NSCs can be isolated directly from the adult brain, primarily from neurogenic niches. Additional adult sources include the olfactory mucosa and the spinal cord ependymal region surrounding the central canal. During development, NSCs and neural progenitors can be derived from fetal brain tissue, particularly from the ventricular zone (VZ) at mid-gestation [141]. Alternatively, NSCs can be generated in vitro from induced pluripotent stem cells (iPSCs) reprogrammed from somatic cells through defined transcription factors (e.g., OCT4, SOX2, KLF4, c-MYC) [142]. Direct lineage reprogramming (transdifferentiation) represents another strategy in which somatic or glial cells are converted into neural lineage cells through neural transcription factors (e.g., ASCL1, BRN2, NEUROD1) [144]. These approaches provide distinct experimental and translational routes for obtaining NSCs, each with specific biological, technical, and ethical considerations.

The translation of NSC-based therapies into clinical settings relies on the scalable and ethical procurement of competent progenitor cells. As schematically described in Figure 4, the biological sources and experimental strategies to obtain NSCs extend from direct anatomical isolation to advanced somatic reprogramming. Historically, endogenous NSCs have been harvested from primary fetal tissues or from adult neurogenic niches and alternative reservoirs such as the olfactory mucosa and spinal cord central canal [139,140]. While these primary sources provide cells with robust inherent neurogenic potential, their clinical application is often obstructed by strict ethical constraints, limited tissue accessibility and poor in vitro scalability [141]. To avoid these translational limitations, cellular engineering has provided revolutionary alternatives (Figure 4). The generation of iPSCs from patient-derived somatic cells, followed by directed neural induction, offers an inexhaustible, autologous source of NSCs [142]. Furthermore, direct lineage reprogramming, or transdifferentiation, allows the rapid conversion of somatic or glial cells directly into neural progenitors or mature neurons by expressing specific neural transcription factors, bypassing the pluripotent state [143,144]. Together, these methodological approaches provide a comprehensive toolkit for disease modeling and regenerative medicine, requiring a careful balance between cell yield, epigenetic stability, and safety.

ESCs are stem cells that are obtained from the embryoblast in embryonic tissue and are considered pluripotent [145]. Despite their potential, other different ways of obtaining NSCs have been developed due to the difficulty of their extraction at this stage. On the other hand, fetal or fetal tissue stem cells, as the name implies, represent pluripotent cells derived from tissue developing during and after the blastocyst stage, so they are known to be more mature than ESCs [146]. Both ESCs and fetal stem cells are only obtained during the developmental stages in mammals, and it is natural to think about the ethical implications of obtaining them in humans, which could be why the cultivation of embryonic cells with only epigenetic differentiation has been proposed for the transplantation of NSC in humans [141]. There are also cells obtained from immediate postnatal models, described as neonatal cells, with a time window for their extraction being usually short [147].

iPSCs are generated from somatic cells [3]; this is perhaps the most significant advance in recent years regarding the NSC and has been developed for the most part by the extensive work of Yamanaka and collaborators [148] thanks to cellular somatic reprogramming. The induction of human iPSCs exposed to neural precursors in a selective medium for proliferation and expansion has allowed the development of a scalable method of culture of NSCs. The induction of iPSCs from human and mouse fibroblasts through certain factors earned Shinya Yamanaka’s team the Nobel Prize recognition. This induction made it possible to identify both NSCs and NPCs as populations that can be derived from differentiated somatic cells. Through this reprogramming process, mature cells are reverted to a multipotent state, granting them the ability to differentiate into diverse neural lineages. This marked the beginning of a new era, between the extraction of NSCs directly from neurogenic brain niches and the ability to provide NSCs from the same organism from which cells such as fibroblasts are extracted, making a more personalized and individual therapy [149]. Although there is no absolute clarity on the factors related to this reprogramming, the use of Oct3/4, Sox2, c-Myc and the Krüppel-like 4 (KLF4) factor seems to work adequately [148]. This induced differentiation could be performed with the intention of generating granular cells in the OB from cells in embryogenesis for postnatal neurogenesis [150]. Some articles suggest that obtaining NSCs for the treatment of chronic diseases in humans seems to have better results when they are based on good practice and disease-derived iPSCs or ESCs for specific diseases rather than those obtained from fetal or primary sources [151]. These types of techniques are more scalable and less limited than those of original tissue, in addition to being specific to the neuronal pathology to be studied or treated, and in the clinic, represent a specific form of treatment for the patient. However, strict control of cell programming is required, and there is a high risk for tumor generation.

We can find the techniques that correspond to transdifferentiation [132], that is, direct reprogramming from somatic cells. This procedure is based on altering the fate of mature somatic cells for conversion to NSCs, avoiding the intermediate pluripotency state of iPSCs. This is achieved by introducing transcription factors of regulatory genes through the use of viral vectors or plasmids, promoting the development of neural phenotyping [152]. At the same time, another approach used for the same purpose is chemical induction with low-weight molecules that alter cell epigenetics and modulate signaling pathways such as TGF-β, modifying the cell’s lineage and avoiding the induction of external genes. Kim et al. [153] initially established this method of direct transdifferentiation from fibroblasts to NPCs, skipping the pluripotency induction that Ymanaka et al. described [153]. Not long after, Thier et al. [144] showed the successful integration of neural cells such as neurons, astrocytes and oligodendrocytes generated by this method into rat brains, validating the technique [144]. In these described cases, the chances of tumor generation are lower than those of iPSCs, as well as the reduction of problems related to immunity, but some cells maintain their epigenetic memory, maintaining the possibility of a reversal to the previous phenotype [152]. In addition, the existing protocols are still premature, and their continued future research is necessary due to low conversion rates reported [154].

### 4.2. Culture Procedures of Adult Neural Stem Cells

**Figure 5:**
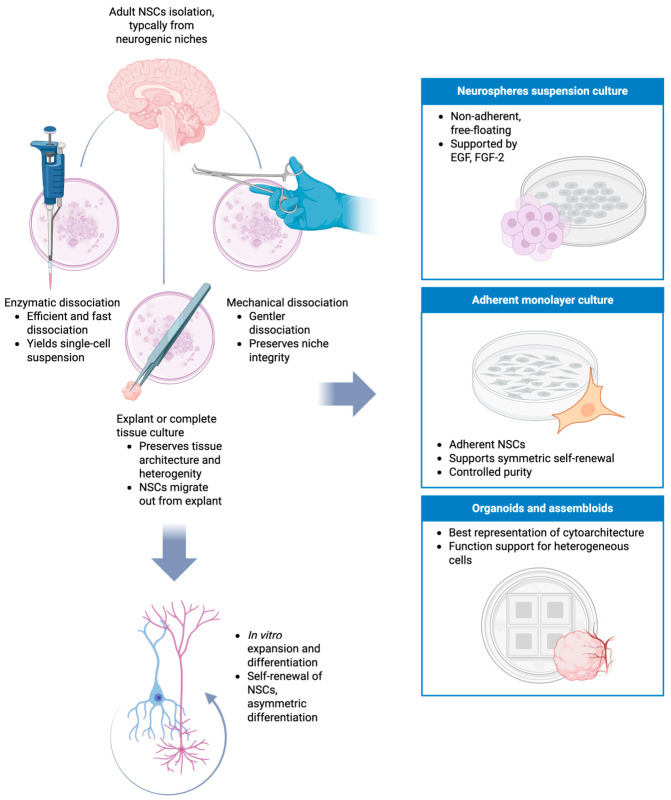
Culture strategies for adult NSCs. Schematic representation of the main in vitro culture methods for adult neural stem cells (NSCs). After isolation from neurogenic regions (typically SVZ or SGZ), tissue can undergo enzymatic or mechanical dissociation to obtain single-cell suspensions or be maintained as explants to preserve aspects of niche structure [57]. Cells may then be expanded in suspension cultures like neurospheres [139] or in adherent monolayer systems [155] supplemented with mitogens such as epidermal growth factor (EGF) and fibroblast growth factor-2 (FGF-2) [157]. Withdrawal or modulation of growth factors promotes lineage-specific differentiation into neurons, astrocytes, or oligodendrocytes [139]. Each approach differs in its impact on cellular heterogeneity, preservation of stem cell properties, and experimental control.

The principal culture strategies used for adult NSCs are summarized in Figure 5. In a general manner, following tissue procurement from neurogenic regions, cells can be obtained through enzymatic or mechanical dissociation to generate single-cell suspensions or, alternatively, through explant culture, which partially preserves niche architecture [155,156]. These initial approaches are followed by expansion under suspension conditions (neurosphere assay) or adherent monolayer systems supplemented with mitogens [157]. Subsequent manipulation of growth factor exposure enables controlled differentiation into neural lineages. Each method presents distinct advantages and technical limitations that influence cellular heterogeneity, maintenance of stemness, and experimental reproducibility, which will be described in the following paragraphs.

One of the first successful cultures in the isolation of undifferentiated neural cells from mice showed nestin expression during the primary stages of culture and subsequently the development of astrocytic cell line morphology. Thus, demonstrating that cells in an in vitro medium could be differentiable [139]. Then, in a study carried out in 1995 [158], it was possible to maintain the culture of NSCs with culture medium enriched with FGF2 for one year. Multiple passages of the culture were performed, and then the cells were inoculated into adult rat brains, and their viability was verified for more than three months after implantation [158]. This finding demonstrated that it is possible to preserve the multipotentiality characteristics of progenitor cells in culture media under the right conditions and, in addition, the usefulness of these cells when successfully transplanted in a live model [158]. The efficiency in the production of NSC continued to be demonstrated, and the evidence amassed in 2003 [159]. A medium supplemented with NS-A and B27 was used in cultures with a density greater than five cells per millimeter. The cells obtained were obtained from adult rat models, commonly from the subependymal zone. In this way, it was possible to culture and isolate cells for more than six months, which had a history of more than 50 cell passages, conserving cell morphology. These initial cultures were already capable of differentiating multipotent precursor cells from astrocytic and oligodendroglial cells, in such a way that the culture of NSCs was demonstrated [122,160]. Also, the difficulty in the growth of the NCSs began to be seen after several passages and throughout the differentiation process, while the asymmetry division through which they went through was described [161]. Since these initial studies, culture media have been supplemented with specific growth and differentiation factors to optimize NSC expansion and lineage commitment. Subsequent evidence demonstrated that NSC cultures exposed to EGF exhibit enhanced proliferation, characterized by highly mitotically active cells expressing DLX2, consistent with type C transit-amplifying progenitors. These EGF-responsive type C cells have been shown to generate neurospheres in cell cultures, indicating that purified C cells retain robust self-renewal and proliferative capacity in vitro [162].

Neurosphere culture refers to the formation of cell clusters (NSCs/NPCs) without adhesion, which allows a three-dimensional growth, closer to the microenvironment present in in vivo models. This culture technique began with the extraction of type B cells from the SVZ of the lateral ventricles in rodents [26]. The use of serum-free medium such as Dulbecco’s modified Eagle Medium (DMEM) and DMEM:F12 enriched with growth media such as EGF, FGF2, heparin and B27 has been used for the culture of NSCs as neurospheres. In these cultures, the preservation of the qualities of multipotentiality, migration and cell differentiation after two weeks of passages and cultures has been demonstrated [27]. The type of neurosphere suspension culture is identified as an effective way to grow NSCs in a self-renewing population while retaining their multipotential properties. Cellular differentiation of NSC has been demonstrated in a culture of neurospheres towards an astrocyte line with exposure to fetal bovine serum, while differentiation towards a neuronal cell line occurs with exposure to factors such as cyclic dibutyryl adenosine monophosphate (cAMP) [163]. The advantages and disadvantages of neurosphere culture in its most direct purposes have been discussed, such as the use of cultures for the growth and study of NSCs and their reproduction and differentiation. One of the main challenges with neurosphere cultures is the fact of heterogeneity, as the results obtained from cells vary according to the method used and the specific region or neurogenic niche from which they are taken [164].

The nature of neurospheres is considered heterogeneous, since these structures are usually composed of a mixture of neural cells in three-dimensional formation in different stages of proliferation and growth, which may include NSCs, NPCs or cells already determined to a specific differentiation. In addition, the cellular composition of these groups can vary depending on the age of the organism from which they are extracted, the seeding density of the cultures, the conditions of the cultures and the number of cell passages [165]. Ultrastructural studies in neurospheres have revealed irregular nuclei in their cells, in particular dependent on the spatial region in which each cell is located within the neurospheres, especially in those more exposed to the surface compared to the innermost part of the structure [166]. They also show intermediate filaments or microtubules and multivesiculated bodies in their cytosol, as well as protrusions in their plasma membrane, indicating intense intercellular communication. Interestingly, scattered cisterns of ringed lamellae have been described in cells that form neurospheres, which are membranous structures that have previously been found in tumor cells and germ cells [166]. However, tumor neurospheres, or gliospheres, usually have a dense network of intermediate filaments and microtubules, which is not observed in non-tumor neurospheres [165].

The approach of technology such as 3D printing on current dilemmas in tissue regeneration and materials science regarding stem cells seems to be an open field for the exploration of possible solutions [167]. The three-dimensional environment of cells in cultures such as organoids favors the preservation of cell identity with much more fidelity than other cultures, mainly because of the opportunity to include microglial cells that allow it [124], since the extracellular matrix is capable of modulating the expression of NSCs and their progenitor derivation [168]. Organoids are three-dimensional tissue models created in vitro and derived from iPSCs [169]; this allows the self-organization and self-regulation capacity of a set of cells to be used by more accurately simulating the tissue microenvironment of an in vivo model [170]. Despite the differences between species, the integration of human tissue organoids into rat brain circuits has recently been demonstrated [171]. This integration turned out to be bidirectional, demonstrating the formation of synapses when receiving direct projections from the thalamus of the animal brain and generating actions after organoid stimulation. This proved that peripheral sensorial stimuli in the rat generated electrical responses within human tissue. At the same time, the authors used optogenetic techniques to activate neurons in the organoid. Stimulation of these human cells induced reward-seeking behaviors in the animals, proving this bidirectionality [171]. However, this technique represents similar challenges to those of neurospheres, and monolayer cultures continue to show some advantages over organoids, as monolayer cultures are usually purer and have faster proliferation rates [172], allowing experimentations that require high-throughput screening, such as those used in drug testing [173].

### 4.3. Characterization of Cell Culture Phentotypes

The laboratory methods used for the characterization of NSCs are diverse and are chosen according to the specific aspects of the cells to be studied. Even so, the capacity for self-renewal and the capacity for cell differentiation in neurons, astrocytes and oligodendrocytes remains one of the few valid methods to identify multipotent NSCs in intensely heterogeneous neurospheres [174]. There is a difference in the markers that are expressed in cells present in neurospheres dependent on their location, since a greater expression of Nes has been observed in cells located in the outer periphery of the neurospheres, in contrast to a greater expression of GFAP in cells located in the center of these. Neurospheres derived from the post-infarction cortex in mice appear to express genes such as the Pax6 and Sox2 genes, while Oct4 has only been observed in ESCs [175]. Similarly, some NSC-specific markers are less expressed in neurosphere cultures compared to cultures with cell adhesion, since the expression of Nes, A2B5, CXCR4, Sox2, Sox8, Sox9 and Ki67 is low in fresh cells and derived from neurospheres and moderate to high in adherent cells [176]. However, for NSCs and NPCs in adult rats isolated from SVZ, the expression of Nes, Sox2 and GFAP in neurospheres was confirmed [177].

Immunocytochemistry is an invaluable tool for cell phenotyping in neurospheres, as it allows the identification of genetic and phenotypic changes resulting from experimental manipulations, as well as the localization of proteins in intact tissues. However, immunocytochemistry by itself does not indicate the percentage of positive cells in a population, so it is recommended to complement it with mRNA quantification. Gene expression analyses, such as real-time polymerase chain reaction (RT-PCR) or qRT-PCR (quantitative), are used to quantify the mRNA expression of stem cell markers and neural lineages in neurospheres [178]. This method allows for accurate molecular analysis and comparison of various expression profiles between different culture conditions or with fresh brain tissue, with accurate quantification of the expression of specific markers. Although it is one of the most widely used and recommended forms for characterization, one of the limitations of this type of analysis is that it does not analyze the percentage of individual cells expressing the marker, so there is a need to also implement methods such as immunohistochemistry or immunofluorescence [32].

The determination of the type of cells based on their morphology and ultrastructure can be performed through transmission electron microscopy (TEM). This is considered a reliable method for the evaluation of the state of neurospheres because it allows the identification of cellular states such as apoptosis, necrosis or subtle structural changes visible in the mitochondria or the endoplasmic reticulum [165]. However, this method can represent a confounding factor for the characterization of cells because the morphology alone in undifferentiated states is not precise, and it has been described that factors such as overstimulation with growth factors end up limiting it [179].

Other types of analysis have been implemented in the cellular characterization of NSCs, such as the Neural Colony Forming Cell Assay (N-CFCA), which is based on the size of the colonies and is able to discriminate between different cell lines, since NSCs exhibit greater proliferative capacity than NPCs [180]. There is also ELDA (Extreme Limiting Dilution Assay) software that is used for quantitative analysis of dilution tests and that allows the generation of logarithmic fraction plots, providing confidence intervals at the same time. FACS and proliferation analysis by labeling with BrdU or EdU are also options for the identification of NSCs. Even more advanced techniques such as single-cell RNA-seq are used, which can reveal the transcriptional signals and activated pathways of cell lineages in NSCs within a neurogenic niche in vivo [181].

On the other hand, cultures like iPSCs require the manual selection of undifferentiated colonies under direct observation, which are identifiable by their non-uniform morphology, and differentiated cell colonies are usually eliminated [182].

### 4.4. Limitations of Current In Vitro and In Vivo Models

While in vitro expansion of NSCs and iPSCs has revolutionized disease modeling, 2D and suspension models possess significant technical limits. A primary limitation of iPSC-derived neural models is the deletion of age-related epigenetic signatures during the reprogramming process. This resets the biological clock of cells, making it difficult to accurately model late-onset neurodegenerative diseases without artificially inducing cellular aging [183]. Also, extended propagation in standard 2D monolayers or neurospheres inherently leads to genomic instability. As mentioned before, studies have shown that long-term cultured neurospheres experience a progressive decline in their differentiation capacity and accumulate genetic aberrations [184,185]. The prolonged expansion of human pluripotent stem cells frequently induces acquired cancer-related mutations and chromosomal abnormalities, raising severe safety concerns for downstream translational applications, being one of the most relevant downsides [186].

To overcome limitations of 2D cultures, 3D cerebral organoids act as powerful models to mimic cytoarchitecture of the human brain. However, they are constrained by the absence of functional vascular networks. The lack of perfusion limits oxygen and nutrient diffusion to approximately 200 to 400 µm, inevitably leading to a necrotic core in mature organoids and limiting their maximum viable size [187]. Single-cell transcriptomic analyses have revealed that the in vitro microenvironment of organoids induces chronic metabolic stress, particularly glycolysis and endoplasmic reticulum stress, which severely impairs proper molecular subtype specification and cortical patterning [188]. Finally, traditional organoid protocols derive only from the neuroectoderm, meaning they inherently lack neuroimmune modulators, such as microglia and endothelial cells, unless these populations are exogenously introduced through complex co-culture systems.

On the other hand, in vivo animal models (predominantly rodents) have provided the foundational understanding of AN and NSC dynamics. Yet, translating these findings to humans remains a great challenge due to profound interspecies disparities. The mammalian brain exhibits distinct evolutionary adaptations, such as the continuous RMS, observed primarily in adult mice, which is virtually absent or restricted in adult humans [189]. Additionally, we know rodents do not naturally develop neurodegenerative conditions like Alzheimer’s disease (AD). Modeling such pathologies relies on forced expression of familial mutated genes that fail to accurately capture the sporadic nature and complex neuroinflammatory microenvironment of human neurodegeneration [190]. Thus, relying exclusively on murine models often overestimates the efficacy of NSC-based interventions, necessitating a combined approach that incorporates advanced human-derived in vitro systems.

## 5. Neural Stem Cell Research in Injury and Disease

Given the close relationship between CNS pathologies and NSC proliferation, several methods and techniques derived from cell culturing have been proposed as potential therapeutic strategies for a variety of related disorders [125,191].

### 5.1. Neurodegenerative Diseases

In the first clinical and pathological description of AD, made by Aloysius [192] at the beginning of the twentieth century, the clinical symptoms of a 51-year-old patient with rapid progressive memory loss that began with disorientation and mood swings, progressing to delirium, confusion, auditory hallucinations and total loss of temporal-spatial recognition were detailed [192]. Histopathological analysis after the patient’s death showed what’s identified as generalized atrophy of brain tissue and, for the first time, the presence of fibrillary glomeruli and accumulation of “a brain waste substance still pending examination,” which today is known as neurofibrillary tangles and the accumulation of Tau protein, respectively [192].

The finding in recent years related to the alteration in hippocampal neurogenesis in adult humans with cognitive problems, caused by either a mild cognitive disparity or advanced neurodegenerative diseases such as AD, has resulted in an important scientific advance [193]. In a study from 2019 [6], the presence and number of immature neural cells in the brains of healthy patients up to the ninth decade of life and patients with AD were compared, finding this type of cell in the brains of all patients. However, the number of cells that could progressively mature in a patient with AD was much lower as the disease progressed [6]. It is also known that the relationship between AN and memory is close [194].

In a study published in 2009 [195], a direct correlation was observed between AN and spatial memory. It was found that specifically blocking the neurogenesis process in the DG of the hippocampus in adult rats resulted in a spatial loss in tests such as the water maze and a loss of recognition of objects dependent on this region, progressively and in the long-term [195]. The conversation involving the concepts of AD, on the one hand, and AN on the other, becomes interesting when trying to explain which of the two phenomena affects the other. A decrease in hippocampal AN has been demonstrated in patients with AD versus patients in similar conditions without disease. However, it had been recently talked about that the decrease in the generation of new neural cells in the adult brain is a contributing factor to the manifestations of AD and not the other way around [196]. Therapies that currently stand out are neural repair with the use of EVs and therapies based on the use of therapeutic applications of CRISPR-Cas9 screening for modifying DNA for therapy for AD, for example [197,198]. Ultimately, problems related to stem cell therapy could benefit from the integration of artificial intelligence models, especially with models of convolutional neural networks as explored by [199].

Subsequently, in an AD model, the paracrine effect of NSCs was identified by the improvement of certain functions in the animal models, such as the improvement of spatial memory and the reduction in expression of inflammation pathways by deactivation of microglia and the secretion of anti-inflammatory factors [200].

AN has also been intensively studied in the context of Parkinson’s disease (PD). This is a neurodegenerative disease, progressive and characterized by the loss of dopaminergic neurons in the substantia nigra of the adult brain, as well as by the formation of Lewis bodies [201]. The non-motor symptoms associated with PD, such as cognitive dysfunction, anxiety, depression or sleep disorders, are present in the early stages of the disease and have been linked to alterations in neurogenesis emphasized in both patients with PD and in animal models [202]. In addition, a significant reduction in SVZ thickness has been reported in adult patients, showing that the density of NSCs also decreases in the same region [203]. One of the pathways involved in reducing neurogenesis in this disease is the Notch1 pathway and its descendant signaling pathways, which appear to lead to an increase in apoptosis and thus a decrease in neuronal survival. This is because of alpha-synuclein (α-syn) and its overexpression, causing aberrant neuronal differentiation [204].

Mutations associated with PD such as D620N, associated with a late and dominant onset of Parkinson’s, are related to the alteration of hippocampal neurogenesis in adult mice. This is reflected by a reduction in the volume of cells, as well as in their proliferation, differentiation and cell migration [201].

Deep brain stimulation, a current treatment for neurodegenerative diseases such as PD or Huntington’s disease, has also been found to promote cell proliferation and neurogenesis in the SVZ by causing an increase in the thickness of this region [203]. Similarly, the use of NSCs in the brain could bring benefits to healthy aging but more importantly, to cognitive alterations linked to diseases related to the elderly [205].

### 5.2. Mood Disorders

Mood disorders, primarily comprising major depressive disorder (MDD) and bipolar disorder, are psychiatric conditions characterized by profound disruptions in emotional state and cognitive function. Extensive evidence links these clinical diagnoses to impairments in adult hippocampal neurogenesis. In patients with MDD, a reduction in hippocampal volume and a decrease in the generation of new neurons are commonly observed [206]. This neurogenic deficit contributes to specific cognitive impairments, such as deficits in pattern separation, which may explain the over-generalization of negative emotions seen in depression [97,207]. Furthermore, the therapeutic efficacy of several antidepressant treatments strongly correlates with their ability to stimulate NSC proliferation and restore neurogenic capacity, highlighting the critical role of new neurons in mood regulation and stress resilience [208,209].

Other disorders such as anxiety have been linked to a decrease in AN [2]. However, it is proposed that such states have a much broader base beyond the deregulation of AN. An example of the above is that not all adult patients with cognitive alterations due to their age suffer from anxiety. One hypothesis that could try to explain this situation is that the process of neurogenesis is involved in these disorders not as an etiological agent but as a temporary state for brain regulation [210].

One of the leading hypotheses of depression is the neurobiological hypothesis, which holds that neurons in the adult brain undergo pathological processes that lead to inadequate mood control and failure of antidepressant treatment [211]. Some studies suggest a decrease in hippocampal volume in patients suffering from depression, and different models have been proposed for the diversity of scientific findings in this regard, contemplating the interaction between the various factors that modify, alter or contribute to the depressive state [212]. The proposal of a theory of the origin of depression related to AN results from the fact that the proliferation of new neural cells under situations of induced stress decreases in DG, which then causes episodes of depression. The neurogenesis process was related as an altered factor in episodes of depression and relevant in their recovery [213].

### 5.3. Neuroinflammation as a Biological Modulator of Adult Neurogenesis

Neuroinflammation underlines the pathophysiology of many neurological and psychiatric conditions, including the aforementioned mood disorders and neurodegenerative diseases. Unlike clinical diagnoses, neuroinflammation is a biological response driven by the activation of resident immune cells, primarily microglia and reactive astrocytes [8]. Under chronic pathological conditions, these cells shift to a pro-inflammatory state, releasing cytokines such as IL-1 β, Interleukin-6 (IL-6), and TNF-α. This inflammatory environment acts as a detrimental modulator of the neurogenic niche, suppressing NSC and progenitor cell proliferation, decreasing the availability of essential neurotrophic factors like BDNF, and triggering cell cycle arrest or apoptosis [214,215]. Consequently, chronic neuroinflammation serves as a key biological mechanism that stops regenerative processes and drives the neurogenic decline observed in both severe depression and neurodegeneration [9].

Exposure to drugs such as non-steroidal anti-inflammatory drugs (NSAIDs) contributes to the restoration of neurogenesis [215]. Along the same lines, the activation of microglia in response to neuroinflammation has been found [216]. In adult rat models, a brain infusion cannula was placed through which lipopolysaccharides were introduced with the intention of inducing brain inflammation [214]. Then, neurogenesis was studied in the SGZ niche in the hippocampus, finding an impairment in proliferative activity after concomitant inflammation. These results were notable for the presence of inflamed cells near the colonies of newly formed cells. Also, a negative correlation was found between the number of new neurons and activated microglia, meaning the response to inflammation in the tissue in the presence of new neural cells affects its growth [214]. On the other hand, in the human SVZ, transcriptomic analyses throughout the lifetime of patients have shown a marked downregulation of genes related to neurogenesis during childhood, with an increase in the expression of genes associated with the immune system and age, suggesting that neuroinflammation could be a key factor in the early quiescence of this region in humans [217].

### 5.4. Traumatic Spinal Cord Injury

One of the most important discoveries about AN and the pathological states of the nervous system was its relationship with spinal cord injuries. In the Cebus monkey, after a hemisection of the spine, brain activation and proliferation of neural cells increased, with a greater inclusion of mitotic markers in these regions [218]. At the same time, protein synthesis was modified, increasing above and below the area of spinal cord injury. An increase in hippocampal and corpus callosum activity, among other brain regions, was also found in the injured models compared to the healthy ones. It was then suggested that the brain was not indifferent to protein synthesis during spinal cord injury and that a minimal regeneration response may occur [218]. One of the main pieces of evidence of cell migration was in fact the proliferation of new neurons directed towards sites of nerve injury, where astrocytes differentiated to participate in scar formation, indicating a stress response by a CNS injury [219].

Replicating NSC microenvironments through transplantation strategies could enable the generation and delivery of cells capable of restoring functions lost due to injury or disease. This therapeutic potential is largely attributed to their remarkable capacity for adaptation, integration and contribution to AN [220]. Because of the ability of NSCs to differentiate into glial cells, several studies have been done to transplant these types of cells into damaged or severed spinal cords. Glial cells promote neural regeneration and connectivity in the injured spinal cord, but they also assist in the treatment of denuded and demyelinated axons and provide trophic support for endogenous cells already in the spinal cord [221]. There is currently evidence that transplantation of multipotent cells to the replacement of CNS cell lines could favor their restoration in neurodegenerative processes [222]. This effect was discovered after the inoculation of NSCs in mouse models of multiple sclerosis, where the migration of these cells was measured and the maturation of the transplanted cells in areas of demyelination was demonstrated, with a decrease in it [9].

The use of iPSCs is deeply studied for cell transplant therapy in spinal cord injuries, with benefits on the immunity of patients, as they are cells derived from their own organism [223]. The forms of inoculation of NSCs in current transplant therapies are: (1) Direct intraspinal injection or directly intracerebral by means of stereotaxis. By this method it is possible to obtain more graft locally, which is why it is generally used in models of neurodegenerative diseases and stroke. However, the placement of the graft directly represents the surgical risk of the procedure and its implications. (2) Intrathecal and intraventricular, in which there is a lower CNS position with less invasiveness and is generally used in amyotrophic lateral sclerosis and multiple sclerosis [131].

It has been recognized that an alternative to cell transplantation is the use of acellular strategies, such as the use of external vesicles or exosomes. The current research opens up the panorama of NSC-derived EVs, nanoparticles that are tropic to brain tissue that release miRs and proteins into stem cells, allowing them to modulate and unleash, by their nature, a low immune response [224]. Although they are a current promise due to their low tumorigenic conversion and their great biocompatibility, they are a tool still under study [225].

### 5.5. Cerebral Vascular Disease

Cerebrovascular disease (CVD) modifies neurogenesis in adults, since an increase in the production of neurons has been observed in the recovery periods of patients with this history [226]. CVD or stroke, which is characterized by interrupting cerebral blood flow and thus causing neuronal death by ischemia, produces a robust neurogenesis response in the adult brain [227]. This type of event produces a cell migration by NSCs to the damaged brain region, with the consequent cell differentiation and establishing new connections in the neuronal circuit and thus contributing to the functional recovery of the tissue [228]. However, the process of endogenous neurogenesis alone is thought to be insufficient for complete and effective brain repair due to the low survival rate of most new cells; more than 80% of migrating neural cells do not survive the first two weeks after injury or fail to differentiate [229].

After an ischemic lesion in rodents, the immature neuroblasts generated in the SVZ left the RMS path to look laterally towards a boundary area to the ischemic region, frequently located in the striatum and cortex [230]. These neuroblasts differentiate into mature neurons, demonstrated as functional GABAergic and cholinergic neurons and with dendrite proliferation in their soma. In this process of migration to the damaged regions, the participation of the tissue environment stands out, specifically the association with the cerebral vasculature [231]. It has also been described that epigenetic mechanisms such as DNA methylation, histone modification or regulation by RNA play an essential role in AN after vascular injury and can be manipulated to improve stroke recovery. miRs such as hsa-miR-124a, hsa-miR-17-92, hsa-miR-146 and hsa-miR-210, among others, seem to be involved in the regulation of this process, being related to the proliferation, differentiation and survival of NSCs at the same time as other factors such as IGF-1, VEGF and EGF [227,232].

Some studies have reported the aberrant formation of new neural cells displaying morphological abnormalities, such as ectopic migration and altered dendritic arborization [233]. These ectopic cells exhibit profound functional alterations, including accelerated and decoupled synaptic connectivity that leads to an aberrant rewiring of the local circuitry [234]. This defective neurogenic response ultimately contributes to the functional impairment and cognitive decline frequently observed after severe brain insults or prolonged periods of hyperexcitability [233].

### 5.6. Translational Difficulties in Neural Stem Cell-Based Therapies

Despite the therapeutic potential demonstrated in preclinical settings, the successful translation of NSC-based therapies into routine clinical practice is hindered by several biological and logistical obstacles. The extremely low survival rate and poor engraftment of transplanted cells is one of these problems. Upon administration, NSCs encounter an adverse, neuroinflammatory microenvironment, often characterized by OS, a lack of neurotrophic support and dense glial scar formation [235]. This severely limits their long-term viability and successful functional integration into preexistent circuits. Closely tied to this challenge is the barrier of the host’s adaptive immune response. Because the vast majority of NSC therapies rely on allogeneic sources, transplanted cells are subject to direct immune recognition due to Major Histocompatibility Complex (MHC) and Human Leukocyte Antigen (HLA) mismatches. Clinical and preclinical evidence has demonstrated that allogeneic neural grafts can trigger severe alloimmunization and subsequent graft rejection [236]. Ensuring long-term graft survival currently mandates the continuous administration of potent systemic immunosuppressants [237], which exposes patients to high risks of opportunistic infections and toxicity. To circumvent this translational obstacle without relying on chronic immunosuppression, bioengineering strategies are actively exploring the generation of hypoimmunogenic or universal donor stem cell lines. By utilizing CRISPR-Cas9-based gene editing to genetically ablate HLA/MHC expression and overexpress immunoinhibitory surface proteins, such as CD47, researchers have successfully engineered pluripotent stem cells and their neural derivatives to evade immune rejection, even in fully immunocompetent allogeneic recipients, both in mice and humans [238,239].

Another critical concern is around the safety of these therapies, particularly regarding tumorigenicity and phenotypic instability, as mentioned before. While pluripotent sources such as iPSCs or ESCs provide scalable origins for NSCs, their incomplete differentiation or acquired genetic aberrations during prolonged in vitro expansion can lead to oncogenic transformation, unwanted epithelial–mesenchymal transitions or teratoma formation in vivo, as described in mouse models by Nori et al. in 2025 [240]. Other reports highlight the need for rigorous toxicological and tumorigenic studies in preclinical trials prior to their use in humans [241].

The transition from laboratory settings to clinical application requires rigorous standardization of cell manufacturing. Producing clinical-grade good manufacturing practice in NSC lines is demanded, as well as quality controls to minimize cellular heterogeneity and ensure a reproducible phenotypic identity across different sets. Addressing these manufacturing limitations and ensuring strict biosafety are current focal points in recent first-in-human phase I clinical trials, which represent the critical frontier for moving NSC transplantation into standard neurotherapeutics, such as in the clinical trials in humans written by Curtis et al. [242] for spinal cord injury and by Leone et al. [243] for progressive multiple sclerosis.

## 6. Concluding Remarks

The current paradigm of neuroscience recognizes that the adult brain retains the plastic capacity for the generation of new neural cells in the CNS known as AN [244], mediated by NSCs mainly found in neurogenic niches such as the SVZ, the SGZ of the hippocampus and the hypothalamus (HVZ) [13]. Research on NSCs has expanded in recent years, allowing a growth in knowledge leading to a clear consensus regarding the mechanism of action of these cells, their involvement in physiology within the context of AN, and their importance within pathological processes such as neurodegenerative diseases, mood disorders or CNS lesions [125]. At the same time, it is recognized that the transition between quiescence, proliferation and differentiation of NSCs into neuronal or glial lineages is regulated by molecular signaling pathways recovered from embryogenic stages, such as Notch, Wnt and SHH [106,245], in addition to mechanisms such as miRs [232]. In addition, the external and internal modulable processes of AN recognized are those of physical exercise and nutritional metabolism in promoting the proliferation of progenitors and rescuing cognitive deficits [89], and chronic stress, inflammation and aging in inducing neurogenic decline by forcing NSCs into deep quiescence or depletion in their reserve [74].

The techniques for obtaining and cultivating NSCs have been developed alongside descriptions of those mechanisms. Isolation techniques by dissection, enzymatic disintegration and cell classification such as FACS have allowed the establishment of both primary cultures and iPSCs [246]. The transition from traditional monolayer cultures to more complex ones such as neurospheres or brain organoids has favored the study of cellular interactions in a way that is closer to that of neurogenic niches and microenvironmental structure [170]. At the same time, the development of iPSCs and the technique of direct reprogramming or transdifferentiation now represent viable methods that overcome previously limiting problems for their study, especially those related to the ethical considerations underlying embryonic or fetal cell cultures, allowing for scalable and potentially autologous cell sources [152]. The convergence of these techniques with other fields of study has allowed the incorporation of techniques such as scRNA-seq, which allows cell heterogeneity to be studied more thoroughly and to adapt new needs to the therapy of grafts and cell transplants [178].

These advances position the use of NSCs in cell therapies as a tool for disease modeling, as well as a promising alternative in the structural and functional repair of degenerated or injured CNS, especially in the context of AD, PD, multiple sclerosis, major depression, spinal cord injury and traumatic brain injury [191]. It is recognized that the therapeutic value of NSCs lies not only in a cell replacement effect but also in the influence that contributes, due to a paracrine effect, to favoring the environment in which they are available for neural regeneration [9,200]. This is done through the secretion of neurotrophic factors such as BDNF and NGF, EVs and immunomodulators, where NSCs manage to attenuate neuroinflammation, promote angiogenesis, rescue synaptic plasticity and modify the hostile microenvironment of injury or degeneration in favor of cell proliferation [197].

Significant knowledge gaps remain in the field, particularly regarding cell heterogeneity. The lack of standardized protocols often leads to suboptimal quality control, yielding cell populations with low purity and variable degrees of maturation. Consequently, predicting the behavior and fate of these cells after transplantation into patients becomes highly challenging [247]. This weakness is also highlighted as one of the main limitations in three-dimensional cell cultures. For this same reason, the characterization of NSCs is also a major challenge, due to an inaccurate molecular load and non-clarity on the signaling pathways in which they underlie being promoted or inhibited, depending on the context and cellular phenotype [197].

A critical factor in the use of NSCs for cell transplantation and in injuries is the survival of the cells after inoculation, since the modulatory reaction of immunity strongly influences their proliferation [248]. It is still not clear how to achieve complete integration of cells into the host, with long-term life and proliferation by synaptic connections to the target neural network. While current techniques are advancing in this area, one of the biggest challenges is their actual functionality within the clinical context [242]. Although proliferated cells in vitro allow us to explore mechanisms and favor the development of the field of neurosciences, their use as therapies still has areas for improvement.

## 7. Future Directions

Future research on NCSs should focus on overcoming the biological and translational barriers that currently limit their clinical feasibility. Integrating recent advancements in bioengineering, gene editing and molecular reprogramming, we propose four critical avenues for future investigation: (1) the engineering of materials that favor the growth of cell cultures while providing a space for the controlled release of neurotrophic factors and anti-inflammatory agents in a way that they can be implemented later in organisms or biological materials [249,250]; (2) the improvement of in vitro models with cell co-cultures for the development of organoids and assembloids to establish vascularized networks, mitigating central necrosis and standardizing cell maturation in such a way to allow the development of study models under more physiologically relevant conditions [251,252]; (3) the new therapies suggest a direction beyond direct cell transplantation with the use of EVs or exosomes derived from NSCs, with which it is possible to avoid risks of immune rejection and tumor formation while transferring neuroprotective loads [225]; and (4) in situ reprogramming and reversing the epigenetic signature of aging to awaken the endogenous quiescent NSC reserve [253].

Therapies must move beyond cell suspensions to overcome the low graft survival rates caused by the hostile, neuroinflammatory microenvironment of the injured CNS. The development of advanced biocompatible materials, such as modifiable granular hydrogels is essential for results. These scaffolds may provide a physical matrix that mimics the extracellular rigidity of the brain and can also be engineered for the controlled, sustained release of neurotrophic factors and anti-inflammatory agents, shielding the graft from local OS and glial scarring [250].

As discussed before, standard 3D organoids are limited by central necrosis and metabolic stress due to the lack of perfusion. Future modeling must prioritize the development of vascularized cerebral organoids and multi-lineage assembloids. By incorporating endothelial networks and exogenous immune populations such as microglia into co-cultures, we can mitigate hypoxic injury and recapitulate the true neurovascular niche. This will allow for the study of neurogenesis and disease mechanisms under more physiological conditions, reducing the gap between rodent models and human pathology [188,252].

To bypass the translational hurdles of tumorigenicity and allogeneic immune rejection, future therapies branch in two promising directions. First, the use of NSC-derived small EVs or exosomes offers a cell-free alternative capable of transferring neuroprotective loads without the risk of oncogenic transformation or immune sensitization [254]. Second, for cell-replacement therapies, the application of CRISPR-Cas9-based gene-editing therapies to engineer a universal donor NSCs by ablating HLA expression and overexpressing CD4 will be needed to eliminate the need for chronic immunosuppression in patients [238,239].

Finally, future paradigms should focus on repairing the brain from within. Rather than relying on exogenous transplantation, refining in situ direct lineage reprogramming represents a safer frontier. Specifically, the use of small-molecule drug cocktails (as chemical transdifferentiation) to modulate pathways like TGF-β/SMAD can efficiently convert endogenous glial scar cells directly into functional neurons or NSCs, avoiding the insertional mutagenesis risks of viral vectors [255]. Coupled with targeted epigenetic rejuvenation to awaken the dormant, aged endogenous NSC pools, these strategies could transform the treatment of late-onset neurodegenerative diseases [253].

The evidence available today, from the molecular biology of neurogenic niches to the bioengineering of structures such as organoids, shows that it is possible to manipulate the mechanisms of CNS plasticity. While the translational challenges associated with graft viability, standardization and the hostility of the pathological microenvironment are significant, the integration of multimodal therapies promises to overcome these barriers. Addressing in an interdisciplinary manner the standardization of protocols and the study of NSCs will help reduce gaps towards the clinical goal of reversing damage in currently relevant neurological, psychiatric and traumatic disorders, improving the prognosis and quality of life of patients all around the world.

## Figures and Tables

**Table 1:** Main cell types involved in mammalian adult neurogenesis with their characteristic molecular markers.

Cell Type	Molecular Markers	Location	Primary Function	Reference
NSCs (Type 1 in the SGZ, Type B in the SVZ)	GFAP, Nestin, Sox2	SGZ in DG of the hippocampus, SVZ of the lateral ventricles	Quiescent and active stem cells, source of continuous production of transit-amplifying cells	Doetsch et al., 1997 [57]; Doetsch et al., 1999 [26]; Suh et al., 2007 [53]
Transient amplifying cells (Type 2 in the SGZ, Type C in the SVZ)	Nestin, TBR2, ASCL1 (Mash1)	SGZ in hippocampal DG, SVZ of the lateral ventricles	Highly proliferative intermediate progenitors, production of neuroblasts.	Kim et al., 2011 [54]; Doetsch et al., 1997 [57]
Neuroblasts (Type 3 in the SGZ, Type A in the SVZ)	DCX, PSA-NCAM	SGZ in the DG hippocampus, SVZ, and migratory tracts such as RMS	Migratory immature neurons transitioning to their integration site	Brown et al., 2003 [28]; Rousselot et al., 1995 [28]; Doetsch et al., 1997 [57]
Mature neurons	NeuN, MAP2	Granule cell layer in DG, OB	Differentiated and synaptically integrated neurons	Mullen et al., 1992 [33]
Astrocytes	GFAP, EGFR, S100β, ALDH1L1	Gray matter, white matter, cerebral cortex and perivascular in the BBB	Responsive to local microenvironment cues, metabolic support and blood–brain barrier maintenance	Gross et al., 1996 [58]; Encinas et al., 2011 [56]
Oligodendrocytes	MAG, Olig2, MOG, NG2, PDGFRa	Axonal myelin and gray matter	CNS axon myelination, trophic and metabolic axonal support, injury-responsive	Menn et al., 2006 [59]; Cai et al., 2007 [55]

NSCs, Neural Stem Cells; SGZ, Subgranular Zone of the Hippocampus; SVZ, Subventricular Zone of the Lateral Ventricles; DG, Dentate Gyrus of the Hippocampus; OB, Olfactory Bulb; GFAP, Glial Fibrillary Acidic Protein; Sox2, SRY-Box 2; TBR2, Brain Protein T-Box-2; ASCL1, Achaete-Scute Transcription Factor of the BHLH Family 1; BBB, Blood–Brain Barrier, RMS, Rostral Migratory Stream; ALDH1L1, Aldehyde Dehydrogenase 1 Family Member L1; ASCL1, Achaete-Scute Family BHLH Transcription Factor 1; NG2, Chondroitin Sulfate Proteoglycan 4; DCX, Doublecortin; S100β, Calcium-Binding Protein B; EGFR, Epidermal Growth Factor Receptor; MAG, Myelin Associated Glycoprotein; MAP2, Microtubule Associated Protein 2; MOG, Myelin Oligodendrocyte Glycoprotein; NeuN, Neuronal nuclear antigen; PDGFRa, Platelet Derived Growth Factor Receptor Alpha; PSA-NCAM, Polysialylated-Neural Cell Adhesion Molecule.

**Table 2:** Main molecular factors involved in adult neurogenesis.

Factor	Function	Brain Area	Cell Line	Signaling Pathway	Impact on Neurogenesis	Observations	Reference
BDNF	Promotes neural survival, differentiation, and maturation	SGZ (hippocampus), SVZ	NSCs, progenitors	TrkB/PI3K-Akt, MAPK	Strong stimulator	Dysregulation associated with depression, Alzheimer’s	Ribeiro & Xapelli, 2021 [105]
Wnt	Promotes neuronal proliferation and differentiation	SGZ, SVZ	Neural progenitors	Wnt/β-catenin	Stimulator	Dysfunction linked to schizophrenia, memory and cancer	Lie et al., 2005 [106]
Notch1	Dysfunction linked to schizophrenia, memory and cancer	SGZ, SVZ	NSCs	Notch intracellular domain (NICD)	Notch intracellular domain (NICD)	Hyperactivity associated with gliomas	Ables et al., 2010 [107]
Cortisol/Glucocorticoids	Suppresses stem cell proliferation	Hippocampus (SGZ)	Stem cells, progenitors	Glucocorticoid (GR) receptors	Inhibitor	High in chronic stress, related to depression	Gould et al., 1992 [108]
Inflammation/Cytokines (e.g., IL-6, TNF-α)	Modulate proliferation and differentiation	SGZ, SVZ	Microglia, neuronal progenitors	NF-κB, JAK/STAT	Variable (context-dependent)	Chronic in Alzheimer’s, Parkinson’s, TBI and epilepsy	Belenguer et al., 2021 [109]
Sox2	Maintains stem cell pluripotency	SGZ, SVZ	NSCs	Internal transcription factors, Notch/RBPJk	Essential for self-renewal	Critical in development and repair; altered in cancer	Ferri et al., 2004 [110]
Estrogens	Promoting proliferation of parents	Hippocampus	Neural progenitors	Estrogen receptors (ERα, ERβ)	Stimulator	Post-menopausal reduction can affect cognition	Tanapat et al., 1999 [111]
FGF2	Stimulates the proliferation of NSCs	SGZ, SVZ	Stem cells and progenitors	FGFR/ERK, PI3K-Akt	Stimulator	Involved in post-injury repair and plasticity	Kuhn et al., 1997 [112]
VEGF	Promotes neurogenesis and angiogenesis	Hippocampus (SGZ)	Endothelial, neuronal stem cells	VEGFR2/PI3K-Akt, MAPK	Stimulator	Relationship with depression and post-stroke recovery	Jin et al., 2002 [113]
SIRT1	Regulates metabolism and cellular longevity	Hippocampus	NSCs	SIRT1/PGC-1α, FOXO	Positive modulator (context-dependent)	Associated with healthy aging and neuroprotection	Prozorovski et al., 2008 [114]

BDNF, brain-derived neurotrophic factor; SGZ, subgranular hippocampal zone; SVZ, subventricular zone of lateral ventricles; TrkB/PI3K-Akt, tropomyosin kinase B receptor activating phosphoinositol 3-kinase pathway; MAPK, mitogen-activated protein kinase; Wnt, Wingless/Integrated signaling protein family; Wnt/β-catenin, β-catenin-dependent Wnt signaling pathway; Notch1, Notch 1 receptor homologous NF-κB, activated B-cell kappa light chain enhancer nuclear factor; IL-6, interleukin-6; TNF-α, tumor necrosis factor-alpha; JAK/STAT, Janus-kinase signaling pathway with signal transducer and transcription activator; TBI, traumatic brain injury; Sox2, SRY-box 2; Erα, estrogen receptor alpha; Erβ, estrogen receptor beta; FGF2, fibroblast growth factor 2; FGFR/ERK, fibroblast growth factor receptor activating signal-regulated extracellular kinase pathway; PI3K-Akt, phosphoinositol 3-kinase pathway; VEGF, vascular endothelial growth factor; VEGFR2/PI3K-Akt, vascular endothelial growth factor 2 regulating phosphoinositol 3-kinase pathway; SIRT1, sirtuin 1; SIRT1/PGC-1α, sirtuin 1 regulating peroxisome proliferator-activated γ receptor 1-alpha coactivator; FOXO, Forkhead box O family of transcription factors.

## Data Availability

No new data were created or analyzed in this study.

## Abbreviations

The following abbreviations are used in this manuscript:

α-syn	Alpha-synuclein
AD	Alzheimer’s disease
ALDH1	Aldehyde dehydrogenase 1 Family Member L1
AN	Adult neurogenesis
ASCL1	Achaete-Scute transcription factor of the BHLH family 1
B2M	Beta-2-microglobulin
BBB	Blood–brain barrier
BDNF	Brain-derived neurotrophic factor
BHLH	Basic helix-loop-helix
Bmal1	Brain and muscle aryl hydrocarbon receptor translocator
BMPs	Bone morphogenic proteins
BrdU	Bromodeoxyuridine
cAMP	Cyclic dibutyryl adenosine monophosphate
CCL11	Eotaxin-1
CD47	Cluster of differentiation 47
CNS	Central nervous system
CVD	Cerebral vascular disease
CX3CR1	CX3C chemokine receptor 1
DCX	Doublecortin
DG	Dentate gyrus
DLX2	Distal-less homeobox 2
DMEM	Dulbecco’s modified Eagle Medium
DOT1L	DOT1-like histone lysine methyltransferase
EBF1	Early B-cell factor 1
EdU	5-ethynyl-2’-deoxyuridine
EGF	Epidermal growth factor
EGFR	Epidermal growth factor receptor
ELDA	Extreme Limiting Dilution Assay
ERα	Estrogen receptor alpha
ERβ	Estrogen receptor beta
ESCs	Embryonic stem cells
EVs	Extracellular vesicles
EZH2	Enhancer of zeste homolog 2
FACS	Fluorescence-activated cell sorting
FASN	Fatty acid synthase
FGF2	Fibroblast growth factor 2
FGFR/ERK	Fibroblast growth factor receptor activating signal-regulated extracellular kinase pathway
FOXO	Forkhead box O family of transcription factors
GABA	Gamma-aminobutyric acid
GALR2	Galanin receptor 2
GFAP	Glial fibrillary acidic protein
GLAST	Glutamate-aspartate transporter
HCD	High-calorie diets
HLA	Human Leukocyte Antigen
HVZ	Hypothalamic ventricular zone
IGF1	Insulin-like growth factor type 1
IL-1	Interleukin-1
IL-6	Interleukin-6
IL12p80	Interleukin 12 p80
iPSCs	Induced pluripotent stem cells
ISL1	ISL LIM homeobox 1
JAK/STAT	Janus-kinase signaling pathway with signal transducer and transcription activator
KLF4	Krüppel-like factor 4
LRIG1	Leucine-rich repeats and immunoglobulin-like domains 1
LVCP	Lateral ventricle choroid plexus
MAG	Myelin-associated glycoprotein
MAP2	Microtubule-associated protein 2
MAPK	Mitogen-activated protein kinase
MBD1	Methyl-CpG binding domain protein 1
MCT8	Monocarboxylate transporter 8
MDD	Major depressive disorder
MEX3A	Mex-3 RNA binding family member A
MHC	Major Histocompatibility Complex
miRs	microRNAs
MOG	Myelin oligodendrocytic glycoprotein
N-CFCA	Neural Colony Forming Cell Assay
NeuN	Neuronal nuclear antigen
NFIA	Nuclear factor 1 A-type
NF-κB	Activated B-cell kappa light chain enhancer nuclear factor
NGF	Nerve growth factor
NGN2	Neurogenin 2
NG2	Chondroitin Sulfate Proteoglycan 4
Notch1	Notch 1 receptor homologous
NPCs	Neural progenitor cells
NPY1R	Neuropeptide Y1 receptor
NSAIDs	Non-steroidal anti-inflammatory drugs
NSCs	Neural stem cells
NT-3	Neurotrophin-3
OATP1C1	Organic anion transporter family member 1C1
OB	Olfactory bulb
Olig2	Oligodendrocytic transcription factor 2
OPCs	Oligodendrocyte precursor cells
OS	Oxidative stress
Pax6	Paired box 6
PD	Parkinson’s disease
PDGFRa	Platelet Derived Growth Factor Receptor Alpha
PER1	Circadian regulator 1
PI3K-Akt	Phosphoinositol 3-kinase pathway
PSA-NCAM	Polysialylated-Neural Cell Adhesion Molecule
PTEN	Phosphatase and tensin homologous protein
qRT-PCR	Quantitative real-time polymerase chain reaction
RGCs	Radial glial cells
RGS6	G-protein signaling regulator 6
RMS	Rostral migratory stream
RNAi	RNA interference
RT-PCR	Real-time polymerase chain reaction
S	Striatum
scRNA-seq	Single-cell RNA sequencing
SGZ	Subgranular zone
SHH	Sonic Hedgehog
SIRT1	Sirtuin 1
SIRT1/PGC-1α	Sirtuin 1 regulating peroxisome proliferator-activated γ receptor 1-alpha coactivator
SIRT7	Sirtuin 7
SN	Substantia nigra
Sox2	SRY-box 2
Sox9	SRY-box 9
Stat3	Signal Transducer and Activator of Transcription 3
SVZ	Subventricular zone
S100β	S100 calcium-binding protein β
TBI	Traumatic brain injury
TBR2	Brain protein T-box 2
TEM	Transmission electron microscopy
TGF-α	Transforming growth factor alpha
TLX (NR2E1)	Nuclear receptor subfamily 2 group E member 1
TNFR1	Tumor necrosis factor receptor 1
TNFR2	Tumor necrosis factor receptor 2
TNF-α	Tumor necrosis factor-alpha
TrkB/PI3K-Akt	Tropomyosin kinase B receptor activating phosphoinositol 3-kinase pathway
VEGF	Vascular endothelial growth factor
VEGFR2/PI3K-Akt	Vascular endothelial growth factor 2 regulating phosphoinositol 3-kinase pathway
Wnt	Wingless/Integrated signaling protein family

## References

[1] Altman J., Das G.D. (1965). Autoradiographic and histological evidence of postnatal hippocampal neurogenesis in rats. J. Comp. Neurol..

[2] Eriksson P.S., Perfilieva E., Björk-Eriksson T., Alborn A.-M., Nordborg C., Peterson D.A., Gage F.H. (1998). Neurogenesis in the adult human hippocampus. Nat. Med..

[3] Gage F.H. (2019). Adult neurogenesis in mammals. Science.

[4] Ming G.-l., Song H. (2011). Adult Neurogenesis in the Mammalian Brain: Significant Answers and Significant Questions. Neuron.

[5] Deng W., Aimone J.B., Gage F.H. (2010). New neurons and new memories: How does adult hippocampal neurogenesis affect learning and memory?. Nat. Rev. Neurosci..

[6] Moreno-Jiménez E.P., Flor-García M., Terreros-Roncal J., Rábano A., Cafini F., Pallas-Bazarra N., Ávila J., Llorens-Martín M. (2019). Adult hippocampal neurogenesis is abundant in neurologically healthy subjects and drops sharply in patients with Alzheimer’s disease. Nat. Med..

[7] Gage F.H., Temple S. (2013). Neural Stem Cells: Generating and Regenerating the Brain. Neuron.

[8] Liddelow S.A., Guttenplan K.A., Clarke L.E., Bennett F.C., Bohlen C.J., Schirmer L., Bennett M.L., Münch A.E., Chung W.-S., Peterson T.C. (2017). Neurotoxic reactive astrocytes are induced by activated microglia. Nature.

[9] Pluchino S., Muzio L., Imitola J., Deleidi M., Alfaro-Cervello C., Salani G., Porcheri C., Brambilla E., Cavasinni F., Bergamaschi A. (2008). Persistent inflammation alters the function of the endogenous brain stem cell compartment. Brain.

[10] Kalamakis G., Brüne D., Ravichandran S., Bolz J., Fan W., Ziebell F., Stiehl T., Catalá-Martinez F., Kupke J., Zhao S. (2019). Quiescence Modulates Stem Cell Maintenance and Regenerative Capacity in the Aging Brain. Cell.

[11] Bonaguidi M.A., Wheeler M.A., Shapiro J.S., Stadel R.P., Sun G.J., Ming G.L., Song H. (2011). In Vivo Clonal Analysis Reveals Self-Renewing and Multipotent Adult Neural Stem Cell Characteristics. Cell.

[12] Gross C.G. (2000). Neurogenesis in the adult brain: Death of a dogma. Nat. Rev. Neurosci..

[13] Sánchez-Gomar I., Geribaldi-Doldán N., Santos-Rosendo C., Sanguino-Caneva C., Carrillo-Chapman C., Fiorillo-Moreno O., Villareal Camacho J.L., Quiroz E.N., Verástegui C. (2024). Exploring the Intricacies of Neurogenic Niches: Unraveling the Anatomy and Neural Microenvironments. Biomolecules.

[14] Quiñones-Hinojosa A., Sanai N., Soriano-Navarro M., Gonzalez-Perez O., Mirzadeh Z., Gil-Perotin S., Romero-Rodriguez R., Berger M.S., Garcia-Verdugo J.M., Alvarez-Buylla A. (2006). Cellular composition and cytoarchitecture of the adult human subventricular zone: A niche of neural stem cells. J. Comp. Neurol..

[15] Dumitru I., Paterlini M., Zamboni M., Ziegenhain C., Giatrellis S., Saghaleyni R., Björklund Å., Alkass K., Tata M., Druid H. (2025). Identification of proliferating neural progenitors in the adult human hippocampus. Science.

[16] Robins S.C., Stewart I., McNay D.E., Taylor V., Giachino C., Goetz M., Ninkovic J., Briancon N., Maratos-Flier E., Flier J.S. (2013). α-Tanycytes of the adult hypothalamic third ventricle include distinct populations of FGF-responsive neural progenitors. Nat. Commun..

[17] Makrygianni E.A., Chrousos G.P. (2023). Neural Progenitor Cells and the Hypothalamus. Cells.

[18] Feliciano D.M., Bordey A., Bonfanti L. (2015). Noncanonical Sites of Adult Neurogenesis in the Mammalian Brain. Cold Spring Harb. Perspect. Biol..

[19] Ernst A., Alkass K., Bernard S., Salehpour M., Perl S., Tisdale J., Possnert G., Druid H., Frisén J. (2014). Neurogenesis in the Striatum of the Adult Human Brain. Cell.

[20] Roeder S.S., Burkardt P., Rost F., Rode J., Brusch L., Coras R., Englund E., Håkansson K., Possnert G., Salehpour M. (2022). Evidence for postnatal neurogenesis in the human amygdala. Commun. Biol..

[21] Mourtzi T., Dimitrakopoulos D., Kakogiannis D., Salodimitris C., Botsakis K., Meri D.K., Anesti M., Dimopoulou A., Charalampopoulos I., Gravanis A. (2021). Characterization of substantia nigra neurogenesis in homeostasis and dopaminergic degeneration: Beneficial effects of the microneurotrophin BNN-20. Stem Cell Res. Ther..

[22] Lie D.C., Dziewczapolski G., Willhoite A.R., Kaspar B.K., Shults C.W., Gage F.H. (2002). The adult substantia nigra contains progenitor cells with neurogenic potential. J. Neurosci..

[23] Sanai N., Nguyen T., Ihrie R.A., Mirzadeh Z., Tsai H.-H., Wong M., Gupta N., Berger M.S., Huang E., Garcia-Verdugo J.-M. (2011). Corridors of migrating neurons in the human brain and their decline during infancy. Nature.

[24] Roubinet C., White I.J., Baum B. (2021). Asymmetric nuclear division in neural stem cells generates sibling nuclei that differ in size, envelope composition, and chromatin organization. Curr. Biol..

[25] Merkle F.T., Mirzadeh Z., Alvarez-Buylla A. (2007). Mosaic Organization of Neural Stem Cells in the Adult Brain. Science.

[26] Doetsch F., Caillé I., Lim D.A., García-Verdugo J.M., Alvarez-Buylla A. (1999). Subventricular Zone Astrocytes Are Neural Stem Cells in the Adult Mammalian Brain. Cell.

[27] Chen K., Hughes S.M., Connor B. (2007). Neural Progenitor Cells Derived from the Adult Rat Subventricular Zone: Characterization and Transplantation. Cell Transplant..

[28] Brown J.P., Couillard-Després S., Cooper-Kuhn C.M., Winkler J., Aigner L., Kuhn H.G. (2003). Transient expression of doublecortin during adult neurogenesis. J. Comp. Neurol..

[29] Lledo P.-M., Merkle F.T., Alvarez-Buylla A. (2008). Origin and function of olfactory bulb interneuron diversity. Trends Neurosci..

[30] Altman J. (1969). Autoradiographic and histological studies of postnatal neurogenesis. IV. Cell proliferation and migration in the anterior forebrain, with special reference to persisting neurogenesis in the olfactory bulb. J. Comp. Neurol..

[31] Garcia A.D.R., Doan N.B., Imura T., Bush T.G., Sofroniew M.V. (2004). GFAP-expressing progenitors are the principal source of constitutive neurogenesis in adult mouse forebrain. Nat. Neurosci..

[32] Codega P., Silva-Vargas V., Paul A., Maldonado-Soto A.R., DeLeo A.M., Pastrana E., Doetsch F. (2014). Prospective Identification and Purification of Quiescent Adult Neural Stem Cells from Their In Vivo Niche. Neuron.

[33] Mullen R.J., Buck C.R., Smith A.M. (1992). NeuN, a neuronal specific nuclear protein in vertebratesxs. Development.

[34] Feng R., Rampon C., Tang Y.-P., Shrom D., Jin J., Kyin M., Sopher B., Martin G.M., Kim S.-H., Langdon R.B. (2001). Deficient Neurogenesis in Forebrain-Specific *Presenilin-1* Knockout Mice Is Associated with Reduced Clearance of Hippocampal Memory Traces. Neuron.

[35] Llorente V., Velarde P., Desco M., Gómez-Gaviro M.V. (2022). Current Understanding of the Neural Stem Cell Niches. Cells.

[36] Englund C., Fink A., Lau C., Pham D., Daza R.A.M., Bulfone A., Kowalczyk T., Hevner R.F. (2005). Pax6, Tbr2, and Tbr1 Are Expressed Sequentially by Radial Glia, Intermediate Progenitor Cells, and Postmitotic Neurons in Developing Neocortex. J. Neurosci..

[37] Sessa A., Ciabatti E., Drechsel D., Massimino L., Colasante G., Giannelli S., Satoh T., Akira S., Guillemot F., Broccoli V. (2016). The Tbr2 Molecular Network Controls Cortical Neuronal Differentiation Through Complementary Genetic and Epigenetic Pathways. Cereb. Cortex.

[38] Clelland C.D., Choi M., Romberg C., Clemenson G.D., Fragniere A., Tyers P., Jessberger S., Saksida L.M., Barker R.A., Gage F.H. (2009). A Functional Role for Adult Hippocampal Neurogenesis in Spatial Pattern Separation. Science.

[39] Kokoeva M.V., Yin H., Flier J.S. (2005). Neurogenesis in the Hypothalamus of Adult Mice: Potential Role in Energy Balance. Science.

[40] Kaplan M.S., McNelly N.A., Hinds J.W. (1985). Population dynamics of adult-formed granule neurons of the rat olfactory bulb. J. Comp. Neurol..

[41] Kempermann G., Kuhn H.G., Gage F.H. (1997). More hippocampal neurons in adult mice living in an enriched environment. Nature.

[42] Lois C., García-Verdugo J.-M., Alvarez-Buylla A. (1996). Chain Migration of Neuronal Precursors. Science.

[43] Peretto P., Merighi A., Fasolo A., Bonfanti L. (1997). Glial Tubes in the Rostral Migratory Stream of the Adult Rat. Brain Res. Bull..

[44] Chazal G., Durbec P., Jankovski A., Rougon G., Cremer H. (2000). Consequences of Neural Cell Adhesion Molecule Deficiency on Cell Migration in the Rostral Migratory Stream of the Mouse. J. Neurosci..

[45] Kaneko N., Marín O., Koike M., Hirota Y., Uchiyama Y., Wu J.Y., Lu Q., Tessier-Lavigne M., Alvarez-Buylla A., Okano H. (2010). New Neurons Clear the Path of Astrocytic Processes for Their Rapid Migration in the Adult Brain. Neuron.

[46] Paredes M.F., James D., Gil-Perotin S., Kim H., Cotter J.A., Ng C., Sandoval K., Rowitch D.H., Xu D., McQuillen P.S. (2016). Extensive migration of young neurons into the infant human frontal lobe. Science.

[47] Sanai N., Tramontin A.D., Quiñones-Hinojosa A., Barbaro N.M., Gupta N., Kunwar S., Lawton M.T., McDermott M.W., Parsa A.T., Manuel-García Verdugo J. (2004). Unique astrocyte ribbon in adult human brain contains neural stem cells but lacks chain migration. Nature.

[48] Shihabuddin L.S., Horner P.J., Ray J., Gage F.H. (2000). Adult Spinal Cord Stem Cells Generate Neurons after Transplantation in the Adult Dentate Gyrus. J. Neurosci..

[49] Seaberg R.M., Smukler S.R., van der Kooy D. (2005). Intrinsic differences distinguish transiently neurogenic progenitors from neural stem cells in the early postnatal brain. Dev. Biol..

[50] Petrik D., Jörgensen S., Eftychidis V., Siebzehnrubl F.A. (2022). Singular Adult Neural Stem Cells Do Not Exist. Cells.

[51] Cebrian-Silla A., Nascimento M.A., Redmond S.A., Mansky B., Wu D., Obernier K., Romero Rodriguez R., Gonzalez-Granero S., García-Verdugo J.M., Lim D.A. (2021). Single-cell analysis of the ventricular-subventricular zone reveals signatures of dorsal and ventral adult neurogenesis. eLife.

[52] Bottes S., Jaeger B.N., Pilz G.-A., Jörg D.J., Cole J.D., Kruse M., Harris L., Korobeynyk V.I., Mallona I., Helmchen F. (2021). Long-term self-renewing stem cells in the adult mouse hippocampus identified by intravital imaging. Nat. Neurosci..

[53] Suh H., Consiglio A., Ray J., Sawai T., D’Amour K.A., Gage F.H. (2007). In Vivo Fate Analysis Reveals the Multipotent and Self-Renewal Capacities of *Sox2*^+^ Neural Stem Cells in the Adult Hippocampus. Cell Stem Cell.

[54] Kim E.J., Ables J.L., Dickel L.K., Eisch A.J., Johnson J.E. (2011). Ascl1 (Mash1) Defines Cells with Long-Term Neurogenic Potential in Subgranular and Subventricular Zones in Adult Mouse Brain. PLoS ONE.

[55] Cai J., Chen Y., Cai W.-H., Hurlock E.C., Wu H., Kernie S.G., Parada L.F., Lu Q.R. (2007). A crucial role for Olig2 in white matter astrocyte development. Development.

[56] Encinas J.M., Michurina T.V., Peunova N., Park J.-H., Tordo J., Peterson D.A., Fishell G., Koulakov A., Enikolopov G. (2011). Division-Coupled Astrocytic Differentiation and Age-Related Depletion of Neural Stem Cells in the Adult Hippocampus. Cell Stem Cell.

[57] Doetsch F., García-Verdugo J.M., Alvarez-Buylla A. (1997). Cellular Composition and Three-Dimensional Organization of the Subventricular Germinal Zone in the Adult Mammalian Brain. J. Neurosci..

[58] Gross R.E., Mehler M.F., Mabie P.C., Zang Z., Santschi L., Kessler J.A. (1996). Bone Morphogenetic Proteins Promote Astroglial Lineage Commitment by Mammalian Subventricular Zone Progenitor Cells. Neuron.

[59] Menn B., Garcia-Verdugo J.M., Yaschine C., Gonzalez-Perez O., Rowitch D., Alvarez-Buylla A. (2006). Origin of Oligodendrocytes in the Subventricular Zone of the Adult Brain. J. Neurosci..

[60] Martins-Macedo J., Araújo B., Anjo S.I., Silveira-Rosa T., Patrício P., Alves N.D., Silva J.M., Teixeira F.G., Manadas B., Rodrigues A.J. (2024). Glial-restricted precursors stimulate endogenous cytogenesis and effectively recover emotional deficits in a model of cytogenesis ablation. Mol. Psychiatry.

[61] Nait-Oumesmar B., Decker L., Lachapelle F., Avellana-Adalid V., Bachelin C., Van Evercooren A.B.- (1999). Progenitor cells of the adult mouse subventricular zone proliferate, migrate and differentiate into oligodendrocytes after demyelination. Eur. J. Neurosci..

[62] Lee D.-C., Chen J.-H., Hsu T.-Y., Chang L.-H., Chang H., Chi Y.-H., Chiu I.-M. (2017). Neural stem cells promote nerve regeneration through IL12-induced Schwann cell differentiation. Mol. Cell. Neurosci..

[63] van Praag H., Kempermann G., Gage F.H. (1999). Running increases cell proliferation and neurogenesis in the adult mouse dentate gyrus. Nat. Neurosci..

[64] Bondolfi L., Ermini F., Long J.M., Ingram D.K., Jucker M. (2004). Impact of age and caloric restriction on neurogenesis in the dentate gyrus of C57BL/6 mice. Neurobiol. Aging.

[65] Leeman D.S., Hebestreit K., Ruetz T., Webb A.E., McKay A., Pollina E.A., Dulken B.W., Zhao X., Yeo R.W., Ho T.T. (2018). Lysosome activation clears aggregates and enhances quiescent neural stem cell activation during aging. Science.

[66] Ali A.A., Schwarz-Herzke B., Stahr A., Prozorovski T., Aktas O., von Gall C. (2015). Premature aging of the hippocampal neurogenic niche in adult Bmal1-deficient mice. Aging.

[67] Du Preez A., Onorato D., Eiben I., Musaelyan K., Egeland M., Zunszain P.A., Fernandes C., Thuret S., Pariante C.M. (2021). Chronic stress followed by social isolation promotes depressive-like behaviour, alters microglial and astrocyte biology and reduces hippocampal neurogenesis in male mice. Brain Behav. Immun..

[68] Valente T., Hidalgo J., Bolea I., Ramirez B., Anglés N., Reguant J., Morelló J.R., Gutiérrez C., Boada M., Unzeta M. (2009). A Diet Enriched in Polyphenols and Polyunsaturated Fatty Acids, LMN Diet, Induces Neurogenesis in the Subventricular Zone and Hippocampus of Adult Mouse Brain. J. Alzheimer’s Dis..

[69] Sorrells S.F., Paredes M.F., Cebrian-Silla A., Sandoval K., Qi D., Kelley K.W., James D., Mayer S., Chang J., Auguste K.I. (2018). Human hippocampal neurogenesis drops sharply in children to undetectable levels in adults. Nature.

[70] Puvogel S., Alsema A., North H.F., Webster M.J., Weickert C.S., Eggen B.J.L. (2024). Single-Nucleus RNA-Seq Characterizes the Cell Types Along the Neuronal Lineage in the Adult Human Subependymal Zone and Reveals Reduced Oligodendrocyte Progenitor Abundance with Age. eNeuro.

[71] Boldrini M., Fulmore C.A., Tartt A.N., Simeon L.R., Pavlova I., Poposka V., Rosoklija G.B., Stankov A., Arango V., Dwork A.J. (2018). Human Hippocampal Neurogenesis Persists throughout Aging. Cell Stem Cell.

[72] Smith L.K., White C.W., Villeda S.A. (2018). The systemic environment: At the interface of aging and adult neurogenesis. Cell Tissue Res..

[73] Bickford P.C., Kaneko Y., Grimmig B., Pappas C., Small B., Sanberg C.D., Sanberg P.R., Tan J., Douglas Shytle R. (2015). Nutraceutical intervention reverses the negative effects of blood from aged rats on stem cells. AGE.

[74] Buckley M.T., Sun E.D., George B.M., Liu L., Schaum N., Xu L., Reyes J.M., Goodell M.A., Weissman I.L., Wyss-Coray T. (2023). Cell-type-specific aging clocks to quantify aging and rejuvenation in neurogenic regions of the brain. Nat. Aging.

[75] Wang C.-L., Ohkubo R., Mu W.-C., Chen W., Fan J.L., Song Z., Maruichi A., Sudmant P.H., Pisco A.O., Dubal D.B. (2023). The mitochondrial unfolded protein response regulates hippocampal neural stem cell aging. Cell Metab..

[76] Vukovic J., Colditz M.J., Blackmore D.G., Ruitenberg M.J., Bartlett P.F. (2012). Microglia Modulate Hippocampal Neural Precursor Activity in Response to Exercise and Aging. J. Neurosci..

[77] Ruetz T.J., Pogson A.N., Kashiwagi C.M., Gagnon S.D., Morton B., Sun E.D., Na J., Yeo R.W., Leeman D.S., Morgens D.W. (2024). CRISPR–Cas9 screens reveal regulators of ageing in neural stem cells. Nature.

[78] Silva-Vargas V., Maldonado-Soto A.R., Mizrak D., Codega P., Doetsch F. (2016). Age-Dependent Niche Signals from the Choroid Plexus Regulate Adult Neural Stem Cells. Cell Stem Cell.

[79] Zhao X., Wang Y., Wait E., Mankowski W., Bjornsson C.S., Cohen A.R., Zuloaga K.L., Temple S. (2021). 3D Image Analysis of the Complete Ventricular-Subventricular Zone Stem Cell Niche Reveals Significant Vasculature Changes and Progenitor Deficits in Males Versus Females with Aging. Stem Cell Rep..

[80] Ma C.-L., Ma X.-T., Wang J.-J., Liu H., Chen Y.-F., Yang Y. (2017). Physical exercise induces hippocampal neurogenesis and prevents cognitive decline. Behav. Brain Res..

[81] Liu P.Z., Nusslock R. (2018). Exercise-Mediated Neurogenesis in the Hippocampus via BDNF. Front. Neurosci..

[82] Soto C., Orihuela L.P., Apostol G., Vivar C. (2025). Running Reverses Chronic Stress-Induced Changes in Serotonergic Modulation of Hippocampal Granule Cells and Altered Behavioural Responses. Eur. J. Neurosci..

[83] Vivar C., Peterson B., Pinto A., Janke E., van Praag H. (2023). Running throughout Middle-Age Keeps Old Adult-Born Neurons Wired. eNeuro.

[84] Fabel K., Fabel K., Tam B., Kaufer D., Baiker A., Simmons N., Kuo C.J., Palmer T.D. (2003). VEGF is necessary for exercise-induced adult hippocampal neurogenesis. Eur. J. Neurosci..

[85] Trico J., Marouane E., Tonetto A., Watabe I., Lapotre A., Tighilet B. (2026). A pilot study on early microgliogenesis following unilateral vestibular neurectomy: A key player in vestibular compensation?. PLoS ONE.

[86] Pofahl M., Nikbakht N., Haubrich A.N., Nguyen T., Masala N., Distler F., Braganza O., Macke J.H., Ewell L.A., Golcuk K. (2021). Synchronous activity patterns in the dentate gyrus during immobility. eLife.

[87] Brandhorst S., Choi I.Y., Wei M., Cheng C.W., Sedrakyan S., Navarrete G., Dubeau L., Yap L.P., Park R., Vinciguerra M. (2015). A Periodic Diet that Mimics Fasting Promotes Multi-System Regeneration, Enhanced Cognitive Performance, and Healthspan. Cell Metab..

[88] Melgar-Locatelli S., de Ceglia M., Mañas-Padilla M.C., Rodriguez-Pérez C., Castilla-Ortega E., Castro-Zavala A., Rivera P. (2023). Nutrition and adult neurogenesis in the hippocampus: Does what you eat help you remember?. Front. Neurosci..

[89] Leiter O., Zhuo Z., Rust R., Wasielewska J.M., Grönnert L., Kowal S., Overall R.W., Adusumilli V.S., Blackmore D.G., Southon A. (2022). Selenium mediates exercise-induced adult neurogenesis and reverses learning deficits induced by hippocampal injury and aging. Cell Metab..

[90] Paul A., Chaker Z., Doetsch F. (2017). Hypothalamic regulation of regionally distinct adult neural stem cells and neurogenesis. Science.

[91] Knobloch M., Braun S.M.G., Zurkirchen L., von Schoultz C., Zamboni N., Araúzo-Bravo M.J., Kovacs W.J., Karalay Ö., Suter U., Machado R.A.C. (2013). Metabolic control of adult neural stem cell activity by Fasn-dependent lipogenesis. Nature.

[92] Bowers M., Liang T., Gonzalez-Bohorquez D., Zocher S., Jaeger B.N., Kovacs W.J., Röhrl C., Cramb K.M.L., Winterer J., Kruse M. (2020). FASN-Dependent Lipid Metabolism Links Neurogenic Stem/Progenitor Cell Activity to Learning and Memory Deficits. Cell Stem Cell.

[93] Kandel P., Semerci F., Mishra R., Choi W., Bajic A., Baluya D., Ma L., Chen K., Cao A.C., Phongmekhin T. (2022). Oleic acid is an endogenous ligand of TLX/NR2E1 that triggers hippocampal neurogenesis. Proc. Natl. Acad. Sci. USA.

[94] Ramosaj M., Madsen S., Maillard V., Scandella V., Sudria-Lopez D., Yuizumi N., Telley L., Knobloch M. (2021). Lipid droplet availability affects neural stem/progenitor cell metabolism and proliferation. Nat. Commun..

[95] Du Preez A., Lefèvre-Arbogast S., Houghton V., de Lucia C., Low D.Y., Helmer C., Féart C., Delcourt C., Proust-Lima C., Pallàs M. (2022). The serum metabolome mediates the concert of diet, exercise, and neurogenesis, determining the risk for cognitive decline and dementia. Alzheimer’s Dement..

[96] Magaquian D., Delgado Ocaña S., Perez C., Banchio C. (2021). Phosphatidylcholine restores neuronal plasticity of neural stem cells under inflammatory stress. Sci. Rep..

[97] Snyder J.S., Soumier A., Brewer M., Pickel J., Cameron H.A. (2011). Adult hippocampal neurogenesis buffers stress responses and depressive behaviour. Nature.

[98] Moreno-Madrid I., Arrabal-Gómez C., Romero-Imbroda J., Díaz-Casares A., Fuxe K., Borroto-Escuela D., Serrano-Castro P., Narváez M. (2025). Intracerebroventricular knockdown of NPY1R disrupts NPY1R-GALR2/TrkB heteroreceptor complexes without affecting neuroplasticity or depressive-like behaviour. J. Psychopharmacol..

[99] Holmes M.M. (2016). Social regulation of adult neurogenesis: A comparative approach. Front. Neuroendocrinol..

[100] Surget A., Belzung C. (2022). Adult hippocampal neurogenesis shapes adaptation and improves stress response: A mechanistic and integrative perspective. Mol. Psychiatry.

[101] Paternina-Die M., Martínez-García M., Martín de Blas D., Noguero I., Servin-Barthet C., Pretus C., Soler A., López-Montoya G., Desco M., Carmona S. (2024). Women’s neuroplasticity during gestation, childbirth and postpartum. Nat. Neurosci..

[102] Chaker Z., Segalada C., Kretz J.A., Acar I.E., Delgado A.C., Crotet V., Moor A.E., Doetsch F. (2023). Pregnancy-responsive pools of adult neural stem cells for transient neurogenesis in mothers. Science.

[103] Borrett M.J., Innes B.T., Jeong D., Tahmasian N., Storer M.A., Bader G.D., Kaplan D.R., Miller F.D. (2020). Single-Cell Profiling Shows Murine Forebrain Neural Stem Cells Reacquire a Developmental State when Activated for Adult Neurogenesis. Cell Rep..

[104] Gantner C.W., Hunt C.P.J., Niclis J.C., Penna V., McDougall S.J., Thompson L.H., Parish C.L. (2021). FGF-MAPK signaling regulates human deep-layer corticogenesis. Stem Cell Rep..

[105] Ribeiro F.F., Xapelli S., Calzà L., Aloe L., Giardino L. (2021). Intervention of Brain-Derived Neurotrophic Factor and Other Neurotrophins in Adult Neurogenesis. Recent Advances in NGF and Related Molecules: The Continuum of the NGF “Saga”.

[106] Lie D.-C., Colamarino S.A., Song H.-J., Désiré L., Mira H., Consiglio A., Lein E.S., Jessberger S., Lansford H., Dearie A.R. (2005). Wnt signalling regulates adult hippocampal neurogenesis. Nature.

[107] Ables J.L., DeCarolis N.A., Johnson M.A., Rivera P.D., Gao Z., Cooper D.C., Radtke F., Hsieh J., Eisch A.J. (2010). Notch1 Is Required for Maintenance of the Reservoir of Adult Hippocampal Stem Cells. J. Neurosci..

[108] Gould E., Cameron H., Daniels D., Woolley C., McEwen B. (1992). Adrenal hormones suppress cell division in the adult rat dentate gyrus. J. Neurosci..

[109] Belenguer G., Duart-Abadia P., Jordán-Pla A., Domingo-Muelas A., Blasco-Chamarro L., Ferrón S.R., Morante-Redolat J.M., Fariñas I. (2021). Adult Neural Stem Cells Are Alerted by Systemic Inflammation through TNF-α Receptor Signaling. Cell Stem Cell.

[110] Ferri A.L.M., Cavallaro M., Braida D., Di Cristofano A., Canta A., Vezzani A., Ottolenghi S., Pandolfi P.P., Sala M., DeBiasi S. (2004). Sox2 deficiency causes neurodegeneration and impaired neurogenesis in the adult mouse brain. Development.

[111] Tanapat P., Hastings N.B., Reeves A.J., Gould E. (1999). Estrogen Stimulates a Transient Increase in the Number of New Neurons in the Dentate Gyrus of the Adult Female Rat. J. Neurosci..

[112] Kuhn H.G., Winkler J., Kempermann G., Thal L.J., Gage F.H. (1997). Epidermal Growth Factor and Fibroblast Growth Factor-2 Have Different Effects on Neural Progenitors in the Adult Rat Brain. J. Neurosci..

[113] Jin K., Zhu Y., Sun Y., Mao X.O., Xie L., Greenberg D.A. (2002). Vascular endothelial growth factor (VEGF) stimulates neurogenesis *in vitro* and *in vivo*. Proc. Natl. Acad. Sci. USA.

[114] Prozorovski T., Schulze-Topphoff U., Glumm R., Baumgart J., Schröter F., Ninnemann O., Siegert E., Bendix I., Brüstle O., Nitsch R. (2008). Sirt1 contributes critically to the redox-dependent fate of neural progenitors. Nat. Cell Biol..

[115] Ali A.A.H., von Gall C. (2022). Adult Neurogenesis under Control of the Circadian System. Cells.

[116] Gilhooley M.J., Pinnock S.B., Herbert J. (2011). Rhythmic expression of per1 in the dentate gyrus is suppressed by corticosterone: Implications for neurogenesis. Neurosci. Lett..

[117] Tamai S.-i., Sanada K., Fukada Y. (2008). Time-of-Day-Dependent Enhancement of Adult Neurogenesis in the Hippocampus. PLoS ONE.

[118] Gensburger C., Labourdette G., Sensenbrenner M. (1987). Brain basic fibroblast growth factor stimulates the proliferation of rat neuronal precursor cells in vitro. FEBS Lett..

[119] Anchan R.M., Reh T.A., Angello J., Balliet A., Walker M. (1991). EGF and TGF-α stimulate retinal neuroepithelial cell proliferation in vitro. Neuron.

[120] Gómez-Pinilla F., Knauer D.J., Nieto-Sampedro M. (1988). Epidermal growth factor receptor immunoreactivity in rat brain. Development and cellular localization. Brain Res..

[121] Morrison R.S., Kornblum H.I., Leslie F.M., Bradshaw R.A. (1987). Trophic Stimulation of Cultured Neurons from Neonatal Rat Brain by Epidermal Growth Factor. Science.

[122] Cattaneo E., McKay R. (1990). Proliferation and differentiation of neuronal stem cells regulated by nerve growth factor. Nature.

[123] Goldman S.A., Luskin M.B. (1998). Strategies utilized by migrating neurons of the postnatal vertebrate forebrain. Trends Neurosci..

[124] Popova G., Soliman S.S., Kim C.N., Keefe M.G., Hennick K.M., Jain S., Li T., Tejera D., Shin D., Chhun B.B. (2021). Human microglia states are conserved across experimental models and regulate neural stem cell responses in chimeric organoids. Cell Stem Cell.

[125] Kamata Y., Isoda M., Sanosaka T., Shibata R., Ito S., Okubo T., Shinozaki M., Inoue M., Koya I., Shibata S. (2021). A robust culture system to generate neural progenitors with gliogenic competence from clinically relevant induced pluripotent stem cells for treatment of spinal cord injury. Stem Cells Transl. Med..

[126] Shi Y., Wang M., Mi D., Lu T., Wang B., Dong H., Zhong S., Chen Y., Sun L., Zhou X. (2021). Mouse and human share conserved transcriptional programs for interneuron development. Science.

[127] Forero A., Pipicelli F., Moser S., Baumann N., Grätz C., Gonzalez Pisfil M., Pfaffl M.W., Pütz B., Kielkowski P., Cernilogar F.M. (2024). Extracellular vesicle-mediated trafficking of molecular cues during human brain development. Cell Rep..

[128] Domingo-Muelas A., Duart-Abadia P., Morante-Redolat J.M., Jordán-Pla A., Belenguer G., Fabra-Beser J., Paniagua-Herranz L., Pérez-Villalba A., Álvarez-Varela A., Barriga F.M. (2023). Post-transcriptional control of a stemness signature by RNA-binding protein MEX3A regulates murine adult neurogenesis. Nat. Commun..

[129] Marqués-Torrejón M.Á., Williams C.A.C., Southgate B., Alfazema N., Clements M.P., Garcia-Diaz C., Blin C., Arranz-Emparan N., Fraser J., Gammoh N. (2021). LRIG1 is a gatekeeper to exit from quiescence in adult neural stem cells. Nat. Commun..

[130] Ciceri G., Baggiolini A., Cho H.S., Kshirsagar M., Benito-Kwiecinski S., Walsh R.M., Aromolaran K.A., Gonzalez-Hernandez A.J., Munguba H., Koo S.Y. (2024). An epigenetic barrier sets the timing of human neuronal maturation. Nature.

[131] Yang M., Liu M., Sánchez Y.F., Avazzadeh S., Quinlan L.R., Liu G., Lu Y., Yang G., O’Brien T., Henshall D.C. (2023). A novel protocol to derive cervical motor neurons from induced pluripotent stem cells for amyotrophic lateral sclerosis. Stem Cell Rep..

[132] Hulme A.J., Maksour S., St-Clair Glover M., Miellet S., Dottori M. (2022). Making neurons, made easy: The use of Neurogenin-2 in neuronal differentiation. Stem Cell Rep..

[133] Luongo C., Butruille L., Sébillot A., Le Blay K., Schwaninger M., Heuer H., Demeneix B.A., Remaud S. (2021). Absence of Both Thyroid Hormone Transporters MCT8 and OATP1C1 Impairs Neural Stem Cell Fate in the Adult Mouse Subventricular Zone. Stem Cell Rep..

[134] Elkabes S., DiCicco-Bloom E., Black I. (1996). Brain microglia/macrophages express neurotrophins that selectively regulate microglial proliferation and function. J. Neurosci..

[135] Liu X., Wang Q., Haydar T.F., Bordey A. (2005). Nonsynaptic GABA signaling in postnatal subventricular zone controls proliferation of GFAP-expressing progenitors. Nat. Neurosci..

[136] Kunoh S., Nakashima H., Nakashima K. (2024). Epigenetic Regulation of Neural Stem Cells in Developmental and Adult Stages. Epigenomes.

[137] Goldman-Rakic P.S. (1999). The physiological approach: Functional architecture of working memory and disordered cognition in schizophrenia. Biol. Psychiatry.

[138] Deepti A., Chackochan B.K., Sadanandan S., Menon A.S., Mohandas K., Vengellur A., Sivan U., Chakrapani P.S.B. (2024). An easy and cost-effective method for the isolation and culturing of neural stem/progenitor cells from the subventricular (SVZ) and dentate gyrus (DG) of adult mouse brain. J. Neurosci. Methods.

[139] Reynolds B.A., Weiss S. (1992). Generation of Neurons and Astrocytes from Isolated Cells of the Adult Mammalian Central Nervous System. Science.

[140] Weiss S., Dunne C., Hewson J., Wohl C., Wheatley M., Peterson A.C., Reynolds B.A. (1996). Multipotent CNS Stem Cells Are Present in the Adult Mammalian Spinal Cord and Ventricular Neuroaxis. J. Neurosci..

[141] Vescovi A.L., Parati E.A., Gritti A., Poulin P., Ferrario M., Wanke E., Frölichsthal-Schoeller P., Cova L., Arcellana-Panlilio M., Colombo A. (1999). Isolation and Cloning of Multipotential Stem Cells from the Embryonic Human CNS and Establishment of Transplantable Human Neural Stem Cell Lines by Epigenetic Stimulation. Exp. Neurol..

[142] Takahashi K., Tanabe K., Ohnuki M., Narita M., Ichisaka T., Tomoda K., Yamanaka S. (2007). Induction of Pluripotent Stem Cells from Adult Human Fibroblasts by Defined Factors. Cell.

[143] Pang Z.P., Yang N., Vierbuchen T., Ostermeier A., Fuentes D.R., Yang T.Q., Citri A., Sebastiano V., Marro S., Südhof T.C. (2011). Induction of human neuronal cells by defined transcription factors. Nature.

[144] Thier M., Wörsdörfer P., Lakes Y.B., Gorris R., Herms S., Opitz T., Seiferling D., Quandel T., Hoffmann P., Nöthen M.M. (2012). Direct Conversion of Fibroblasts into Stably Expandable Neural Stem Cells. Cell Stem Cell.

[145] Zholudeva L.V., Jin Y., Qiang L., Lane M.A., Fischer I., Amini S., White M.K. (2021). Preparation of Neural Stem CellsNeural stem cells and Progenitors: Neuronal Production and GraftingGrafting Applications. Neuronal Cell Culture: Methods and Protocols.

[146] Radoszkiewicz K., Jezierska-Woźniak K., Waśniewski T., Sarnowska A. (2023). Understanding Intra- and Inter-Species Variability in Neural Stem Cells’ Biology Is Key to Their Successful Cryopreservation, Culture, and Propagation. Cells.

[147] Harley-Troxell M.E., Dhar M. (2023). Assembling Spheroids of Rat Primary Neurons Using a Stress-Free 3D Culture System. Int. J. Mol. Sci..

[148] Takahashi K., Yamanaka S. (2006). Induction of Pluripotent Stem Cells from Mouse Embryonic and Adult Fibroblast Cultures by Defined Factors. Cell.

[149] Lee D.-H., Lee E.C., Lee J.y., Lee M.R., Shim J.-w., Oh J.S. (2024). Neuronal Cell Differentiation of iPSCs for the Clinical Treatment of Neurological Diseases. Biomedicines.

[150] Merkle F.T., Fuentealba L.C., Sanders T.A., Magno L., Kessaris N., Alvarez-Buylla A. (2014). Adult neural stem cells in distinct microdomains generate previously unknown interneuron types. Nat. Neurosci..

[151] Nie L., Yao D., Chen S., Wang J., Pan C., Wu D., Liu N., Tang Z. (2023). Directional induction of neural stem cells, a new therapy for neurodegenerative diseases and ischemic stroke. Cell Death Discov..

[152] Fernandez-Muñoz B., Garcia-Delgado A.B., Arribas-Arribas B., Sanchez-Pernaute R. (2021). Human Neural Stem Cells for Cell-Based Medicinal Products. Cells.

[153] Kim J., Efe J.A., Zhu S., Talantova M., Yuan X., Wang S., Lipton S.A., Zhang K., Ding S. (2011). Direct reprogramming of mouse fibroblasts to neural progenitors. Proc. Natl. Acad. Sci. USA.

[154] Zhang W.-J., Zhang J. (2025). Application and current challenges of neural stem cells transplantation in clinical trials of spinal cord injury. Int. J. Surg..

[155] Conti L., Pollard S.M., Gorba T., Reitano E., Toselli M., Biella G., Sun Y., Sanzone S., Ying Q.-L., Cattaneo E. (2005). Niche-Independent Symmetrical Self-Renewal of a Mammalian Tissue Stem Cell. PLoS Biol..

[156] Pollard S.M., Conti L., Sun Y., Goffredo D., Smith A. (2006). Adherent Neural Stem (NS) Cells from Fetal and Adult Forebrain. Cereb. Cortex.

[157] Gritti A., Parati E., Cova L., Frolichsthal P., Galli R., Wanke E., Faravelli L., Morassutti D., Roisen F., Nickel D. (1996). Multipotential stem cells from the adult mouse brain proliferate and self-renew in response to basic fibroblast growth factor. J. Neurosci..

[158] Gage F.H., Coates P.W., Palmer T.D., Kuhn H.G., Fisher L.J., Suhonen J.O., Peterson D.A., Suhr S.T., Ray J. (1995). Survival and differentiation of adult neuronal progenitor cells transplanted to the adult brain. Proc. Natl. Acad. Sci. USA.

[159] Wachs F.-P., Couillard-Despres S., Engelhardt M., Wilhelm D., Ploetz S., Vroemen M., Kaesbauer J., Uyanik G., Klucken J., Karl C. (2003). High Efficacy of Clonal Growth and Expansion of Adult Neural Stem Cells. Lab. Investig..

[160] Lois C., Alvarez-Buylla A. (1993). Proliferating subventricular zone cells in the adult mammalian forebrain can differentiate into neurons and glia. Proc. Natl. Acad. Sci. USA.

[161] Gobbel G.T., Choi S.-J., Beier S., Niranjan A. (2003). Long-term cultivation of multipotential neural stem cells from adult rat subependyma. Brain Res..

[162] Doetsch F., Petreanu L., Caille I., Garcia-Verdugo J.-M., Alvarez-Buylla A. (2002). EGF Converts Transit-Amplifying Neurogenic Precursors in the Adult Brain into Multipotent Stem Cells. Neuron.

[163] Ludwig P.E., Patil A.A. (2019). Adult neural stem cell isolation from whole rat brain and neurosphere culture and differentiation. Bratisl. Lek. Listy.

[164] Parmar M., Sjöberg A., Björklund A., Kokaia Z. (2003). Phenotypic and molecular identity of cells in the adult subventricular zone: In vivo and after expansion in vitro. Mol. Cell. Neurosci..

[165] Gil-Perotín S., Duran-Moreno M., Cebrián-Silla A., Ramírez M., García-Belda P., García-Verdugo J.M. (2013). Adult Neural Stem Cells From the Subventricular Zone: A Review of the Neurosphere Assay. Anat. Rec..

[166] Bez A., Corsini E., Curti D., Biggiogera M., Colombo A., Nicosia R.F., Pagano S.F., Parati E.A. (2003). Neurosphere and neurosphere-forming cells: Morphological and ultrastructural characterization. Brain Res..

[167] Juraski A.C., da Silva V.A., Sharma R., Azzoni A.R., Willerth S.M. (2025). Investigation of novel carboxymethyl chitosan-based bioinks for 3D bioprinting of neural tissues. Biomed. Mater..

[168] Shabani Z., Ghadiri T., Karimipour M., Sadigh-Eteghad S., Mahmoudi J., Mehrad H., Farhoudi M. (2021). Modulatory properties of extracellular matrix glycosaminoglycans and proteoglycans on neural stem cells behavior: Highlights on regenerative potential and bioactivity. Int. J. Biol. Macromol..

[169] Velikic G., Maric D.M., Maric D.L., Supic G., Puletic M., Dulic O., Vojvodic D. (2024). Harnessing the Stem Cell Niche in Regenerative Medicine: Innovative Avenue to Combat Neurodegenerative Diseases. Int. J. Mol. Sci..

[170] Gerasimova E., Beenen A.C., Kachkin D., Regensburger M., Zundler S., Blumenthal D.B., Lutzny-Geier G., Winner B., Prots I. (2025). Novel co-culture model of T cells and midbrain organoids for investigating neurodegeneration in Parkinson’s disease. Npj Park. Dis..

[171] Revah O., Gore F., Kelley K.W., Andersen J., Sakai N., Chen X., Li M.-Y., Birey F., Yang X., Saw N.L. (2022). Maturation and circuit integration of transplanted human cortical organoids. Nature.

[172] Sloan S.A., Andersen J., Pașca A.M., Birey F., Pașca S.P. (2018). Generation and assembly of human brain region–specific three-dimensional cultures. Nat. Protoc..

[173] Langhans S.A. (2018). Three-Dimensional in Vitro Cell Culture Models in Drug Discovery and Drug Repositioning. Front. Pharmacol..

[174] Reekmans K., Praet J., Daans J., Reumers V., Pauwels P., Van der Linden A., Berneman Z.N., Ponsaerts P. (2012). Current Challenges for the Advancement of Neural Stem Cell Biology and Transplantation Research. Stem Cell Rev. Rep..

[175] Nakagomi T., Taguchi A., Fujimori Y., Saino O., Nakano-Doi A., Kubo S., Gotoh A., Soma T., Yoshikawa H., Nishizaki T. (2009). Isolation and characterization of neural stem/progenitor cells from post-stroke cerebral cortex in mice. Eur. J. Neurosci..

[176] Behnan J., Stangeland B., Langella T., Finocchiaro G., Tringali G., Meling T.R., Murrell W. (2017). Identification and characterization of a new source of adult human neural progenitors. Cell Death Dis..

[177] Jaberi R., Mirsadeghi S., Kiani S. (2021). In vitro characterization of subventricular zone isolated neural stem cells, from adult monkey and rat brain. Mol. Biol. Rep..

[178] Kalinina A., Lagace D. (2022). Single-Cell and Single-Nucleus RNAseq Analysis of Adult Neurogenesis. Cells.

[179] Lobo M.V., Alonso F.J., Redondo C., López-Toledano M.A., Caso E., Herranz A.S., Paíno C.L., Reimers D., Bazán E. (2003). Cellular characterization of epidermal growth factor-expanded free-floating neurospheres. J. Histochem. Cytochem..

[180] Roy N.S., Wang S., Jiang L., Kang J., Benraiss A., Harrison-Restelli C., Fraser R.A.R., Couldwell W.T., Kawaguchi A., Okano H. (2000). In vitro neurogenesis by progenitor cells isolated from the adult human hippocampus. Nat. Med..

[181] Siebzehnrubl F.A., Vedam-Mai V., Azari H., Reynolds B.A., Deleyrolle L.P., Filippi M.-D., Geiger H. (2011). Isolation and Characterization of Adult Neural Stem Cells. Stem Cell Migration: Methods and Protocols.

[182] D’Aiuto L., Zhi Y., Kumar Das D., Wilcox M.R., Johnson J.W., McClain L., MacDonald M.L., Di Maio R., Schurdak M.E., Piazza P. (2014). Large-scale generation of human iPSC-derived neural stem cells/early neural progenitor cells and their neuronal differentiation. Organogenesis.

[183] Cornacchia D., Studer L. (2017). Back and forth in time: Directing age in iPSC-derived lineages. Brain Res..

[184] Vukicevic V., Jauch A., Dinger T.C., Gebauer L., Hornich V., Bornstein S.R., Ehrhart-Bornstein M., Müller A.M. (2010). Genetic instability and diminished differentiation capacity in long-term cultured mouse neurosphere cells. Mech. Ageing Dev..

[185] Jensen J.B., Parmar M. (2006). Strengths and limitations of the neurosphere culture system. Mol. Neurobiol..

[186] Lezmi E., Jung J., Benvenisty N. (2024). High prevalence of acquired cancer-related mutations in 146 human pluripotent stem cell lines and their differentiated derivatives. Nat. Biotechnol..

[187] McMurtrey R.J. (2016). Analytic Models of Oxygen and Nutrient Diffusion, Metabolism Dynamics, and Architecture Optimization in Three-Dimensional Tissue Constructs with Applications and Insights in Cerebral Organoids. Tissue Eng. Part. C Methods.

[188] Bhaduri A., Andrews M.G., Mancia Leon W., Jung D., Shin D., Allen D., Jung D., Schmunk G., Haeussler M., Salma J. (2020). Cell stress in cortical organoids impairs molecular subtype specification. Nature.

[189] Pollen A.A., Bhaduri A., Andrews M.G., Nowakowski T.J., Meyerson O.S., Mostajo-Radji M.A., Di Lullo E., Alvarado B., Bedolli M., Dougherty M.L. (2019). Establishing Cerebral Organoids as Models of Human-Specific Brain Evolution. Cell.

[190] Eaton S.L., Wishart T.M. (2017). Bridging the gap: Large animal models in neurodegenerative research. Mamm. Genome.

[191] Abud E.M., Ramirez R.N., Martinez E.S., Healy L.M., Nguyen C.H.H., Newman S.A., Yeromin A.V., Scarfone V.M., Marsh S.E., Fimbres C. (2017). iPSC-Derived Human Microglia-like Cells to Study Neurological Diseases. Neuron.

[192] Stelzmann R.A., Norman Schnitzlein H., Reed Murtagh F. (1995). An english translation of alzheimer’s 1907 paper, “über eine eigenartige erkankung der hirnrinde”. Clin. Anat..

[193] Terreros-Roncal J., Moreno-Jiménez E.P., Flor-García M., Rodríguez-Moreno C.B., Trinchero M.F., Cafini F., Rábano A., Llorens-Martín M. (2021). Impact of neurodegenerative diseases on human adult hippocampal neurogenesis. Science.

[194] Terranova J.I., Ogawa S.K., Kitamura T. (2019). Adult hippocampal neurogenesis for systems consolidation of memory. Behav. Brain Res..

[195] Jessberger S., Clark R.E., Broadbent N.J., Clemenson G.D., Consiglio A., Lie D.C., Squire L.R., Gage F.H. (2009). Dentate gyrus-specific knockdown of adult neurogenesis impairs spatial and object recognition memory in adult rats. Learn. Mem..

[196] Geigenmüller J.N., Tari A.R., Wisloff U., Walker T.L. (2024). The relationship between adult hippocampal neurogenesis and cognitive impairment in Alzheimer’s disease. Alzheimers Dement..

[197] Moretti E.H., Lin A.L.Y., Peruzzotti-Jametti L., Pluchino S., Mozafari S. (2025). Neural Stem Cell-Derived Extracellular Vesicles for Advanced Neural Repair. J. Neurochem..

[198] He C., Chen B., Yan C., Zhou X. (2025). Stem cell and CRISPR/Cas9 gene editing technology in Alzheimer’s disease therapy: From basic research to clinical innovation. Front. Genome Ed..

[199] Ramakrishna R.R., Abd Hamid Z., Wan Zaki W.M.D., Huddin A.B., Mathialagan R. (2020). Stem cell imaging through convolutional neural networks: Current issues and future directions in artificial intelligence technology. PeerJ.

[200] Lee I.S., Jung K., Kim I.S., Lee H., Kim M., Yun S., Hwang K., Shin J.E., Park K.I. (2015). Human neural stem cells alleviate Alzheimer-like pathology in a mouse model. Mol. Neurodegener..

[201] Jiang M., Tu H.T., Zhang K., Zhang W., Yu W.P., Xu J., Tan E.K., Guo K.H., Zeng L. (2021). Impaired neurogenesis in the hippocampus of an adult VPS35 mutant mouse model of Parkinson’s disease through interaction with APP. Neurobiol. Dis..

[202] Huber S.J., Paulson G.W., Shuttleworth E.C. (1988). Relationship of motor symptoms, intellectual impairment, and depression in Parkinson’s disease. J. Neurol. Neurosurg. Psychiatry.

[203] Snapyan M., Desmeules F., Munro J., Bérard M., Saikali S., Gould P.V., Richer M., Pourcher E., Langlois M., Dufresne A.M. (2025). Adult Neurogenesis in the Subventricular Zone of Patients with Huntington’s and Parkinson’s Diseases and following Long-Term Treatment with Deep Brain Stimulation. Ann. Neurol..

[204] Desplats P., Spencer B., Crews L., Pathel P., Morvinski-Friedmann D., Kosberg K., Roberts S., Patrick C., Winner B., Winkler J. (2012). α-Synuclein induces alterations in adult neurogenesis in Parkinson disease models via p53-mediated repression of Notch1. J. Biol. Chem..

[205] Navarro Negredo P., Yeo R.W., Brunet A. (2020). Aging and Rejuvenation of Neural Stem Cells and Their Niches. Cell Stem Cell.

[206] Boldrini M., Santiago A.N., Hen R., Dwork A.J., Rosoklija G.B., Tamir H., Arango V., John Mann J. (2013). Hippocampal granule neuron number and dentate gyrus volume in antidepressant-treated and untreated major depression. Neuropsychopharmacology.

[207] Sahay A., Scobie K.N., Hill A.S., O’Carroll C.M., Kheirbek M.A., Burghardt N.S., Fenton A.A., Dranovsky A., Hen R. (2011). Increasing adult hippocampal neurogenesis is sufficient to improve pattern separation. Nature.

[208] Malberg J.E., Eisch A.J., Nestler E.J., Duman R.S. (2000). Chronic antidepressant treatment increases neurogenesis in adult rat hippocampus. J. Neurosci..

[209] Santarelli L., Saxe M., Gross C., Surget A., Battaglia F., Dulawa S., Weisstaub N., Lee J., Duman R., Arancio O. (2003). Requirement of hippocampal neurogenesis for the behavioral effects of antidepressants. Science.

[210] Revest J.M., Dupret D., Koehl M., Funk-Reiter C., Grosjean N., Piazza P.V., Abrous D.N. (2009). Adult hippocampal neurogenesis is involved in anxiety-related behaviors. Mol. Psychiatry.

[211] Tartt A.N., Mariani M.B., Hen R., Mann J.J., Boldrini M. (2022). Dysregulation of adult hippocampal neuroplasticity in major depression: Pathogenesis and therapeutic implications. Mol. Psychiatry.

[212] Eisch A.J., Petrik D. (2012). Depression and hippocampal neurogenesis: A road to remission?. Science.

[213] Jacobs B.L., van Praag H., Gage F.H. (2000). Adult brain neurogenesis and psychiatry: A novel theory of depression. Mol. Psychiatry.

[214] Ekdahl C.T., Claasen J.H., Bonde S., Kokaia Z., Lindvall O. (2003). Inflammation is detrimental for neurogenesis in adult brain. Proc. Natl. Acad. Sci. USA.

[215] Monje M.L., Toda H., Palmer T.D. (2003). Inflammatory blockade restores adult hippocampal neurogenesis. Science.

[216] Wu A., Zhang J. (2023). Neuroinflammation, memory, and depression: New approaches to hippocampal neurogenesis. J. Neuroinflamm..

[217] Lupo G. (2023). Adult neurogenesis and aging mechanisms: A collection of insights. Sci. Rep..

[218] Bernstein J.J., Wells M.R. (1977). Amino acid incorporation in medulla, pons, midbrain and cortex following spinal cord hemisection in the cebus monkey (*Cebus apella*). Brain Res..

[219] Johansson C.B., Momma S., Clarke D.L., Risling M., Lendahl U., Frisén J. (1999). Identification of a neural stem cell in the adult mammalian central nervous system. Cell.

[220] Ourednik V., Ourednik J. (2007). Plasticity of the central nervous system and formation of “auxiliary niches” after stem cell grafting: An essay. Cell Transplant..

[221] Davies J.E., Huang C., Proschel C., Noble M., Mayer-Proschel M., Davies S.J. (2006). Astrocytes derived from glial-restricted precursors promote spinal cord repair. J. Biol..

[222] Kondo T., Funayama M., Tsukita K., Hotta A., Yasuda A., Nori S., Kaneko S., Nakamura M., Takahashi R., Okano H. (2014). Focal transplantation of human iPSC-derived glial-rich neural progenitors improves lifespan of ALS mice. Stem Cell Rep..

[223] Song B., Cha Y., Ko S., Jeon J., Lee N., Seo H., Park K.J., Lee I.H., Lopes C., Feitosa M. (2020). Human autologous iPSC-derived dopaminergic progenitors restore motor function in Parkinson’s disease models. J. Clin. Investig..

[224] Yeo H., Kim Y.J., Seok J., Kwak Y., Jang S.B., Lim N.H., Song K., Lee J., Cho M.C., Kim S.W. (2025). Correction: Therapeutic potential of NGF-enriched extracellular vesicles in modulating neuroinflammation and enhancing peripheral nerve remyelination. Acta Neuropathol. Commun..

[225] Zou Z., Yang X., An G., Shi Z., Wang K., You C. (2025). miR-218a-5p derived from neural stem cell-exosomes inhibits ferroptosis in spinal cord injury through the Bmil/Mettl3/Alox12 axis. Int. J. Biol. Macromol..

[226] Thored P., Arvidsson A., Cacci E., Ahlenius H., Kallur T., Darsalia V., Ekdahl C.T., Kokaia Z., Lindvall O. (2006). Persistent production of neurons from adult brain stem cells during recovery after stroke. Stem Cells.

[227] Liu X., Fan B., Chopp M., Zhang Z. (2020). Epigenetic Mechanisms Underlying Adult Post Stroke Neurogenesis. Int. J. Mol. Sci..

[228] Darsalia V., Heldmann U., Lindvall O., Kokaia Z. (2005). Stroke-induced neurogenesis in aged brain. Stroke.

[229] Nagase T., Kin K., Yasuhara T. (2023). Targeting Neurogenesis in Seeking Novel Treatments for Ischemic Stroke. Biomedicines.

[230] Arvidsson A., Collin T., Kirik D., Kokaia Z., Lindvall O. (2002). Neuronal replacement from endogenous precursors in the adult brain after stroke. Nat. Med..

[231] Ohab J.J., Fleming S., Blesch A., Carmichael S.T. (2006). A neurovascular niche for neurogenesis after stroke. J. Neurosci..

[232] Favaloro F., DeLeo A.M., Delgado A.C., Doetsch F. (2022). miR-17∼92 exerts stage-specific effects in adult V-SVZ neural stem cell lineages. Cell Rep..

[233] Ammothumkandy A., Ravina K., Wolseley V., Tartt A.N., Yu P.N., Corona L., Zhang N., Nune G., Kalayjian L., Mann J.J. (2022). Altered adult neurogenesis and gliogenesis in patients with mesial temporal lobe epilepsy. Nat. Neurosci..

[234] Lybrand Z.R., Goswami S., Zhu J., Jarzabek V., Merlock N., Aktar M., Smith C., Zhang L., Varma P., Cho K.O. (2021). A critical period of neuronal activity results in aberrant neurogenesis rewiring hippocampal circuitry in a mouse model of epilepsy. Nat. Commun..

[235] Peruzzotti-Jametti L., Bernstock J.D., Vicario N., Costa A.S.H., Kwok C.K., Leonardi T., Booty L.M., Bicci I., Balzarotti B., Volpe G. (2018). Macrophage-Derived Extracellular Succinate Licenses Neural Stem Cells to Suppress Chronic Neuroinflammation. Cell Stem Cell.

[236] Krystkowiak P., Gaura V., Labalette M., Rialland A., Remy P., Peschanski M., Bachoud-Lévi A.C. (2007). Alloimmunisation to donor antigens and immune rejection following foetal neural grafts to the brain in patients with Huntington’s disease. PLoS ONE.

[237] McGinley L.M., Chen K.S., Mason S.N., Rigan D.M., Kwentus J.F., Hayes J.M., Glass E.D., Reynolds E.L., Murphy G.G., Feldman E.L. (2022). Monoclonal antibody-mediated immunosuppression enables long-term survival of transplanted human neural stem cells in mouse brain. Clin. Transl. Med..

[238] Deuse T., Hu X., Gravina A., Wang D., Tediashvili G., De C., Thayer W.O., Wahl A., Garcia J.V., Reichenspurner H. (2019). Hypoimmunogenic derivatives of induced pluripotent stem cells evade immune rejection in fully immunocompetent allogeneic recipients. Nat. Biotechnol..

[239] Xu H., Wang B., Ono M., Kagita A., Fujii K., Sasakawa N., Ueda T., Gee P., Nishikawa M., Nomura M. (2019). Targeted Disruption of HLA Genes via CRISPR-Cas9 Generates iPSCs with Enhanced Immune Compatibility. Cell Stem Cell.

[240] Nori S., Okada Y., Nishimura S., Sasaki T., Itakura G., Kobayashi Y., Renault-Mihara F., Shimizu A., Koya I., Yoshida R. (2015). Long-term safety issues of iPSC-based cell therapy in a spinal cord injury model: Oncogenic transformation with epithelial-mesenchymal transition. Stem Cell Rep..

[241] Garitaonandia I., Gonzalez R., Christiansen-Weber T., Abramihina T., Poustovoitov M., Noskov A., Sherman G., Semechkin A., Snyder E., Kern R. (2016). Neural Stem Cell Tumorigenicity and Biodistribution Assessment for Phase I Clinical Trial in Parkinson’s Disease. Sci. Rep..

[242] Curtis E., Martin J.R., Gabel B., Sidhu N., Rzesiewicz T.K., Mandeville R., Van Gorp S., Leerink M., Tadokoro T., Marsala S. (2018). A First-in-Human, Phase I Study of Neural Stem Cell Transplantation for Chronic Spinal Cord Injury. Cell Stem Cell.

[243] Leone M.A., Gelati M., Profico D.C., Gobbi C., Pravatà E., Copetti M., Conti C., Abate L., Amoruso L., Apollo F. (2023). Phase I clinical trial of intracerebroventricular transplantation of allogeneic neural stem cells in people with progressive multiple sclerosis. Cell Stem Cell.

[244] Leal-Galicia P., Chávez-Hernández M.E., Mata F., Mata-Luévanos J., Rodríguez-Serrano L.M., Tapia-de-Jesús A., Buenrostro-Jáuregui M.H. (2021). Adult Neurogenesis: A Story Ranging from Controversial New Neurogenic Areas and Human Adult Neurogenesis to Molecular Regulation. Int. J. Mol. Sci..

[245] Favaro R., Valotta M., Ferri A.L.M., Latorre E., Mariani J., Giachino C., Lancini C., Tosetti V., Ottolenghi S., Taylor V. (2009). Hippocampal development and neural stem cell maintenance require Sox2-dependent regulation of Shh. Nat. Neurosci..

[246] Iqbal M.A., Fong B.C., Slack R.S. (2022). Direct FACS Isolation of Neural Stem/Progenitor Lineages from the Adult Brain. Methods Mol. Biol..

[247] Elkabetz Y., Panagiotakos G., Al Shamy G., Socci N.D., Tabar V., Studer L. (2008). Human ES cell-derived neural rosettes reveal a functionally distinct early neural stem cell stage. Genes Dev..

[248] Darsalia V., Allison S.J., Cusulin C., Monni E., Kuzdas D., Kallur T., Lindvall O., Kokaia Z. (2011). Cell number and timing of transplantation determine survival of human neural stem cell grafts in stroke-damaged rat brain. J. Cereb. Blood Flow. Metab..

[249] Wang J., Chu R., Ni N., Nan G. (2020). The effect of Matrigel as scaffold material for neural stem cell transplantation for treating spinal cord injury. Sci. Rep..

[250] Tigner T.J., Dampf G., Tucker A., Huang Y.C., Jagrit V., Clevenger A.J., Mohapatra A., Raghavan S.A., Dulin J.N., Alge D.L. (2024). Clickable Granular Hydrogel Scaffolds for Delivery of Neural Progenitor Cells to Sites of Spinal Cord Injury. Adv. Healthc. Mater..

[251] Shin M., Ha T., Lee S., Li C., Choi J.H., Choi J.W. (2025). Biohybrid motor neuron spheroid composed of graphene/HUVEC/neural cell for 3D biosensing system to evaluate drug of amyotrophic lateral sclerosis. Nano Converg..

[252] Li Y., Sun X.Y., Zeng P.M., Luo Z.G. (2025). Neural Responses to Hypoxic Injury in a Vascularized Cerebral Organoid Model. Neurosci. Bull..

[253] Li M., Guo H., Carey M., Huang C. (2024). Transcriptional and epigenetic dysregulation impairs generation of proliferative neural stem and progenitor cells during brain aging. Nat. Aging.

[254] Peruzzotti-Jametti L., Bernstock J.D., Willis C.M., Manferrari G., Rogall R., Fernandez-Vizarra E., Williamson J.C., Braga A., van den Bosch A., Leonardi T. (2021). Neural stem cells traffic functional mitochondria via extracellular vesicles. PLoS Biol..

[255] Hu W., Qiu B., Guan W., Wang Q., Wang M., Li W., Gao L., Shen L., Huang Y., Xie G. (2015). Direct Conversion of Normal and Alzheimer’s Disease Human Fibroblasts into Neuronal Cells by Small Molecules. Cell Stem Cell.

